# Cutting-edge patient-specific surgical plates for computer-assisted mandibular reconstruction: The art of matching structures and holes in precise surgery

**DOI:** 10.3389/fsurg.2023.1132669

**Published:** 2023-03-09

**Authors:** Renshun Liu, Yuxiong Su, Jingya Pu, Chunyu Zhang, Weifa Yang

**Affiliations:** ^1^ Shien-Ming Wu School of Intelligent Engineering, South China University of Technology, Guangzhou, China; ^2^ Oral and Maxillofacial Surgery, Faculty of Dentistry, The University of Hong Kong, Prince Philip Dental Hospital, Hong Kong SAR, China; ^3^ Guangzhou Janus Biotechnology Co., Ltd, Guangzhou, China

**Keywords:** patient-specific surgical plates, mandible reconstruction, surgical techniques, computer-assisted surgery, 3D printing

## Abstract

**Objectives:**

Cutting-edge patient-specific surgical plates (PSSPs) are supposed to improve the efficiency, precision, and functional outcomes of mandibular reconstruction. This study characterized the premium role of PSSPs in precise surgery and explored their working principles in computer-assisted mandibular reconstruction (CAMR).

**Methods:**

The PSSPs-enhanced surgical precision was investigated through the model surgery and representative cases. Spatial deviations of reconstruction were characterized by comparing the reconstructed mandible with the virtually designed mandible. Working principles of PSSPs were distinguished by a review of evolving surgical techniques in CAMR.

**Results:**

In the model surgery, spatial deviations between the virtually planned mandible and the reconstructed mandible were 1.03 ± 0.43 mm in absolute distance deviation, 1.70 ± 1.26 mm in intercondylar length, and 1.86 ± 0.91 mm in intergonial length in the study group of PSSPs, significantly smaller than in the control group of conventional prebent surgical plates. Meanwhile, in the study group, distance deviations were 0.51 ± 0.19 mm in bone-plate distance and 0.56 ± 0.28 mm in drilled screw holes, indicating the art of matching structures and holes. The PSSPs-enhanced CAMR was further demonstrated in three representative cases of mandibular reconstruction. Finally, four primary techniques of CAMR were summarized based on a review of 8,672 articles. The premium role of PSSPs was distinguished by the benefits of matching structures and holes.

**Conclusions:**

The PSSPs-enhanced surgical precision was verified through the model surgery and demonstrated in human surgery. Compared to other surgical techniques of CAMR, PSSPs contributed to the precise surgery by the art of matching structures and holes.

## 1. Introduction

The fibula has been a golden choice for mandibular reconstruction since its first report in the 1980s (1). However, the optimal reconstruction of mandible remains challenging due to the complex aesthetic and functional aspects of mandible. Technically, it is also demanding to completely restore the profile of mandible because of the unmatched anatomical structures between mandible and fibula (2). In recent decades, computer-assisted mandibular reconstruction (CAMR) has been developed to enable virtual surgical planning (VSP) and simplify fibular shaping and contouring (3). Multiple patient-specific devices have been developed to transfer VSP to the operation theatre, including rapid-prototyped mandible models, cutting guides, positioning guides, and cutting-edge patient-specific surgical plates (PSSPs).

PSSPs have flourished since the late 2010s because of the rapid development of titanium 3D printing (4). Traditionally, off-the-shelf surgical plates are used for mandibular reconstruction, which shall be manually contoured in congruence with skeletal structures. Manual bending and twisting of surgical plates are technique-sensitive, time-consuming, and prone to empirical errors, unavoidably interfering with surgical outcomes. On the contrary, PSSPs can navigate the precise folding and fixation of bone segments due to the customized 3D architecture (5). Since there is no plate bending, PSSPs can also improve the predictability and repeatability of precise mandibular reconstruction, streamlining the complex surgical procedures (6).

Based on recent studies, PSSPs can likely improve the efficiency, precision, and functional outcomes of CAMR (7). High surgical precision can be achieved even in complex clinical scenarios and among cases with multiple dental implants (8–10). However, it has not been confirmed whether PSSPs can improve surgical precision compared to conventional prebent plates. In addition, the working principles of PSSPs in precise reconstruction have not been clarified, particularly in the context of CAMR. Lastly, the benefits of PSSPs over other techniques should be distinguished to promote PSSPs in the state-of-the-art clinical workflow of digital dentistry.

Therefore, this study characterized the PSSPs-enhanced CAMR through surgery in rapid-prototyped mandible models, by which adverse effects of soft tissue interference and surgeon experience could be avoided. The workflow of PSSPs-enhanced CAMR was also demonstrated in representative human cases of mandibular reconstruction. Finally, the premium role of PSSPs was distinguished by a review of surgical techniques in CAMR.

## 2. Materials and methods

### 2.1. Study design

**Figure 1:**
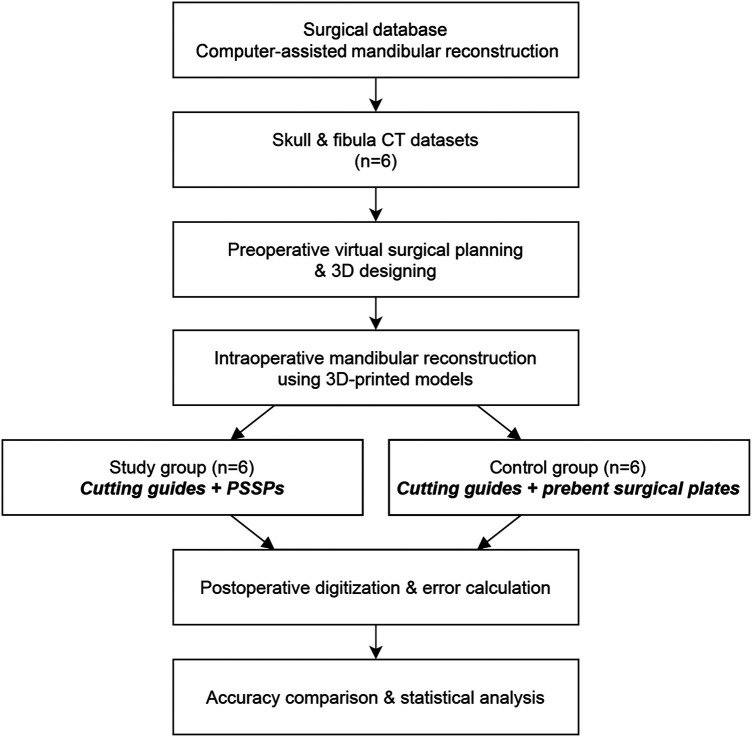
Schematic of study design.

The study design of model surgery is shown in Figure 1. All human data were derived from the Division of Oral and Maxillofacial Surgery at our dental hospital. All procedures complied with the Helsinki Declaration. Ethical approval and informed consents have been obtained. Ethical approval was approved by the Official Review Board with a reference number of UW 16–315.

### 2.2. Model surgery platform

The original Computed Tomography (CT) data of included patients were retrieved from the medical database in our center. CT scan was routinely taken using spiral CT with the tube voltage of 140 KVp, table speed of 40 mm/rotation, gantry tilt of 0°, and slice thickness of less than 1.0 mm. All six patients were diagnosed with primary oral squamous cell carcinoma on the right side. According to the surgical plan, mandibular resection was carried out, followed by the fibular flap reconstruction. According to Brown's classification (11), there were five ipsilateral mandibular defects (class I or II) and one anterior defect (class III), serving as a preliminary test of the impact of different defects. The rapid-prototyped models of mandible and fibula were fabricated by the Stereolithography (SLA) 3D printing (SLA 600, SimpNeed, Hangzhou, China) in polycarbonate resin (SimpNeed), which is a cost-effective material for model surgery with adequate mechanical strength and stability. All models were prepared in double sets for the model surgery of CAMR (Figure 1). One set of models was assigned to the study group of PSSPs, and the other set was assigned to the control group of prebent surgical plates.

### 2.3. Virtual surgical planning and 3d printing

**Figure 2:**
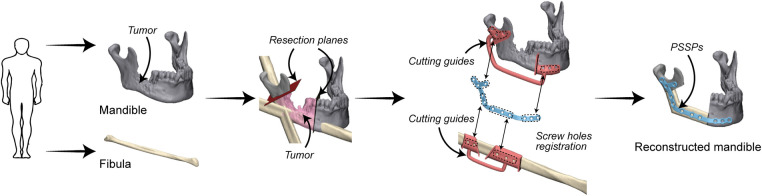
Virtual surgical planning and the workflow of PSSPs-enhanced CAMR.

**Figure 3:**
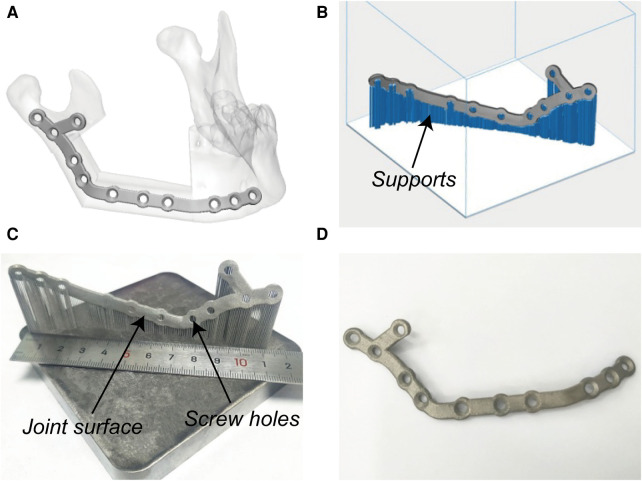
Virtual designing and additive manufacturing of PSSPs. (A) Virtual PSSPs. (B) Design supports. (C) Printed PSSPs with reasonable joint surface and screw holes. (D) Post-processed PSSPs.

Preoperative VSP was conducted in 3-matic 13.0 (Materialise, Leuven, Belgium). Surgical plans of mandibular resection and reconstruction were determined by an experienced surgeon (Prof. Su). Patient-specific devices were designed by the first author (Mr Liu) as previously reported (12), including cutting guides and PSSPs (Figure 2). In brief, PSSPs were designed by drawing a smooth plate and placing screw holes along the outer surface of the mandible. Cutting guides were generated by wrapping the bone surface where the guides would be mounted on. The width and thickness of PSSPs were 6 mm and 2 mm, respectively. Cutting guides were rapid-prototyped in polycarbonate using the SLA 600 printer, whilst PSSPs were manufactured using a metal printer (DiMetal-100, Laseradd, Guangzhou, China) (Figure 3). For PSSPs-enhanced CAMR, screw holes in cutting guides corresponded to those in PSSPs.

PSSPs were additively manufactured with pure titanium particles (Avimetal Powder Metallurgy Technology, Beijing, China). The median diameter of titanium particles was 0.034 mm. Before 3D printing, the digital files of PSSPs were imported into Materialise Magics 21.0 (Materialise) for part orientation, support generation, and model slicing. The sliced PSSPs were then sent to the metal printer for Selective Laser Melting (SLM) with a layer thickness of 0.03 mm, scanning velocity of 500 mm/s, and laser power of 170 W. The metal printing was carried out under an argon atmosphere. After 3D printing, all porous supports were manually removed, and PSSPs were polished and cleansed before application.

### 2.4. PSSPs-enhanced CAMR

**Figure 4:**
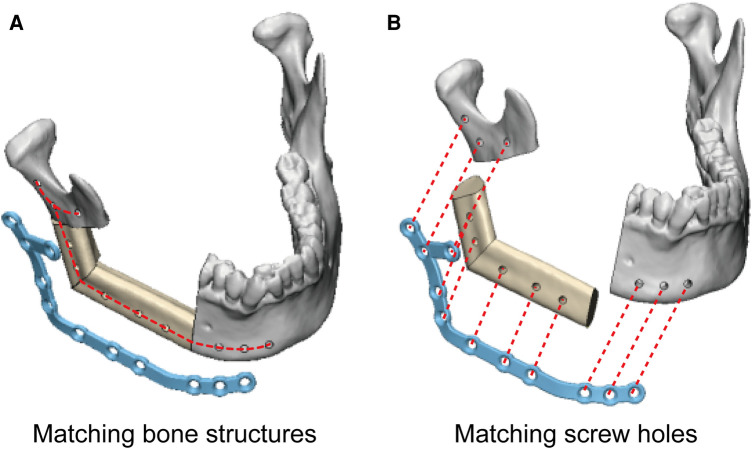
The art of matching structures (A) and holes (B) in PSSPs-enhanced CAMR.

In the study group, the PSSPs-enhanced CAMR were elucidated in Figure 2. The model surgery was performed by a surgeon (Dr. Yang) and an assistant (Mr. Liu). Mandibular resection and fibular osteotomy were performed using a surgical reciprocating saw, assisted by the patient-specific cutting guides. After releasing the cutting guides, screw holes drilled *in situ* could guide the accurate placement of PSSPs. During mandibular reconstruction, fibular segments were folded and fixed to PSSPs according to the customized 3D architecture and matched screw holes. The “fibula-PSSP” complex was transferred to the defect site for mandibular reconstruction. The relative position of mandibular remnants, fibular segments, and PSSPs were determined by the art of matching structures and screw holes, which is shown in Figure 4. For matching structures, PSSPs were designed per the reconstructed mandible, by which PSSPs adapted precisely to the bone contour and provided little tolerance for errors. For matching screw holes, the arrangement of bone segments and PSSPs was facilitated by the rigid registration of screw holes predesigned in VSP. The synergistic matching structures and holes contributed to the precise CAMR.

### 2.5. CAMR with conventional surgical plates

In the control group, off-the-shelf straight surgical plates were made of pure titanium and had the same dimensions as PSSPs. Straight surgical plates were manually prebent based on 3D-printed reconstructed mandible models before surgery. Mandibular resection and fibular osteotomy were also assisted by patient-specific cutting guides, whereas screw holes were not drilled to guide the relative position of prebent surgical plates. Bone segments were carefully aligned and fixed with prebent surgical plates. Surgery largely depended on trial and error and the repeated adjustment between bone segments and the prebent surgical plate.

### 2.6. Model digitization and spatial deviations

**Figure 5:**
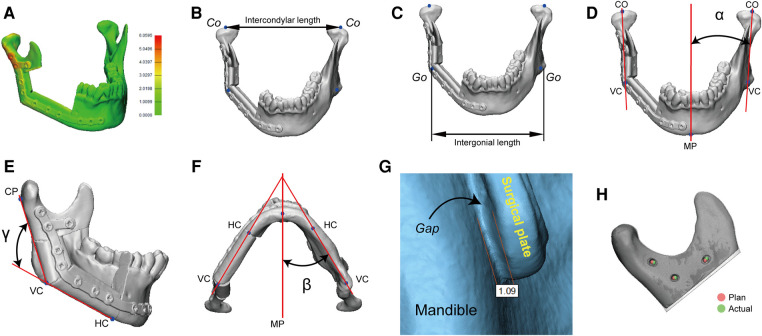
A panel of measurements for surgical precision. Co, condylion; Go, gonion; VC, vertical corner; MP, midsagittal plane; HC, horizontal corner; CP, condylion posterior; *α*, coronal mandibular angle; *β*, axial mandibular angle; *γ*, sagittal mandibular angle.

The reconstructed mandible models were digitized using a high-resolution desktop 3D scanner (EinScan Pro 2X, Shining 3D, Hangzhou, China). Thereafter, the reconstructed mandible was superimposed onto the virtually designed mandible for precision analysis. Two authors (Dr. Yang and Mr. Liu) conducted the precision analysis. 3D spatial deviations were characterized by the following measurements in 3-matic 13.0 (Figure 5) (13–15).

(1) Absolute distance deviation (Figure 5A): the average absolute distance between each point on the postoperative reconstructed mandible to its closest point on the preoperative virtually designed mandible.
(2) Intercondylar length (Figure 5B): the distance difference between the preoperative and postoperative intercondylar lines.
(3) Intergonial length (Figure 5C): the distance difference between the preoperative and postoperative intergonial lines.
(4) Coronal mandibular angle (Figure 5D): the angular difference between the preoperative and postoperative coronal mandibular angles.
(5) Sagittal mandibular angle (Figure 5E): the angular difference between the preoperative and postoperative sagittal mandibular angles.
(6) Axial mandibular angle (Figure 5F): the angular difference between the preoperative and postoperative axial mandibular angles.
(7) Bone-plate distance (Figure 5G): the maximum distance between surgical plate and bone, which quantifies the matching structure.
(8) Screw hole deviation (Figure 5H): the average distance between preoperative and postoperative screw holes, which quantifies the matching screw holes.

### 2.7. Clinical workflow of PSSPs-enhanced CAMR

PSSPs-enhanced CAMR was illustrated in real surgery, so as to demonstrate the art of matching structures and holes. Specific procedures of VSP and 3D printing were as above. Surgery was operated by an experienced surgeon (Prof. Su). The perioperative management was carried out in a routine manner.

### 2.8. A review of CAMR

A review was conducted to investigate the role of PSSPs in the context of CAMR. The detailed methods were included in the Supplementary Materials. In brief, a comprehensive literature search was conducted in PubMed using different combinations of the following keywords: “mandible” and “reconstruction”. All articles published up to August, 2022 were reviewed. A strict broad-to-narrow selection was carried out to identify surgical techniques of CAMR. CAMR was defined as mandibular reconstruction assisted by computer technology, especially for the preoperative VSP, with or without hardware or devices to transfer the VSP to surgery. The inclusion criteria were: (1) VSP was performed for surgery; (2) mandibular reconstruction was performed for defects resulting from primary or secondary mandibular resection. The exclusion criteria were: (1) trauma, orthognathic surgery, or dentoalveolar surgery; (2) without VSP, or the surgical planning was not virtually conducted in computer; (3) not human studies; (4) surgical techniques were not identified in the context. After article selection by two authors (Dr. Yang and Mr. Liu), different surgical techniques were extracted for categorization and analysis. The type and number of hardware or devices used in CAMR were investigated.

### 2.9. Statistics

Continuous data were checked for the normality of distribution before statistical analysis. Spatial deviations of CAMR between the study group and control group were compared using nonparametric tests in SPSS (version 24.0, IBM, Chicago, USA). The threshold for statistical significance was defined as the *p*-value less than 0.05 without adjustment for multiple comparisons. The number of published surgical techniques was plotted against the year of publication to determine the evolution of CAMR.

## 3. Results

Before the model surgery, rapid-prototyped mandible and fibula models were checked with sufficient quality (Supplementary Materials). Spatial deviations of CAMR in model surgery were detailed in Table 1. Absolute distance deviations were 1.03 ± 0.43 mm in the study group and 2.14 ± 0.86 mm in the control group, which showed a significant difference (*p* = 0.02). There were significant differences in intercondylar length, intergonial length, and coronal and sagittal mandibular angles on the operated side. In addition, the bone-plate distance was 0.51 ± 0.19 mm in the study group compared to 0.78 ± 0.32 mm in the control group, indicating a better congruence between PSSPs and the neo-mandible. The distance deviation was 0.56 ± 0.28 mm for screw holes in the study group. Please refer to the Supplementary Materials for relative errors of the condylar head and mandibular angle points in *X*, *Y*, and *Z* directions, again including error data for each case.

**Table 1:** Spatial deviations of CAMR in the study and control groups.

Parameters	Study group (*n* = 6)	Control group (*n* = 6)	*p* value
Absolute distance deviation (mm)	1.03 (0.43)	2.14 (0.86)	0.02*
Intercondylar length (mm)	1.70 (1.26)	4.81 (1.49)	0.004*
Intergonial length (mm)	1.86 (0.91)	5.14 (2.09)	0.01*
Operated side			
Coronal mandibular angle (°)	1.82 (1.00)	4.20 (1.82)	0.02*
Sagittal mandibular angle (°)	2.75 (1.28)	6.52 (2.22)	0.02*
Axial mandibular angle (°)	2.24 (1.46)	5.29 (3.28)	0.13
**Non-operated side**			
Coronal mandibular angle (°)	1.58 (0.81)	2.78 (1.78)	0.18
Sagittal mandibular angle (°)	1.84 (0.44)	3.52 (2.73)	0.31
Axial mandibular angle (°)	1.42 (0.58)	3.09 (2.45)	0.31
Bone-plate distance (mm)	0.51(0.19)	0.78(0.32)	0.001*
Screw hole deviation (mm)	0.56(0.28)	NA	NA

**Figure 6:**
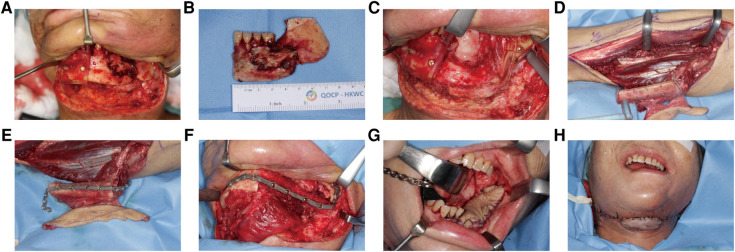
A 65-year-old female with osteoradionecrosis of left mandible underwent PSSPs-enhanced CAMR. (**A**) After exposure, left mandible showed eroded bone surface and pathologic fracture. Cutting guides were mounted to guide mandibular resection. (**B**) The resected bone specimen was shown. (**C**) The defect site was shown, and cutting guides were fixed *in situ* by screws. Resection margins were accurately located by the presence of bleeding bone. (**D**) The left fibular osteo-cutaneous flap was harvested with the cutting guide. (**E**) The fibula was fixed to PSSP using predrilled screw holes. (**F**) The “fibula-plate” was transferred to the defect site and fixed according to predrilled screw holes. (**G**) The intraoral skin island flap was viable. (**H**). The extraoral skin island flap was viable. The neo-mandible contour is satisfying.

**Figure 7:**
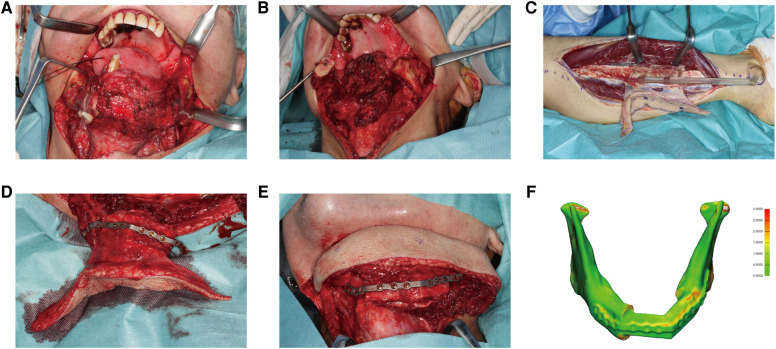
A 67-year-old male presented with squamous cell carcinoma at the tongue and left mandible. (**A**) 3D-printed cutting guides fitted onto the mandible as planned. (**B**) The mandible defect was shown after glossectomy and segmental mandibulectomy. (**C**) The fibula free flap was harvested with the cutting guide. (**D**) The PSSP was fixed onto fibular segments with corresponding screw holes. (**E**) The “fibula-plate” was transferred to the defect site and fixed to corresponding screw holes in the mandibular stumps. (**F**) The reconstructed mandible was superimposed onto the virtually designed mandible. The spatial deviation was visualized with a color map. The absolute distance deviation was 0.57 ± 0.46 mm.

**Figure 8:**
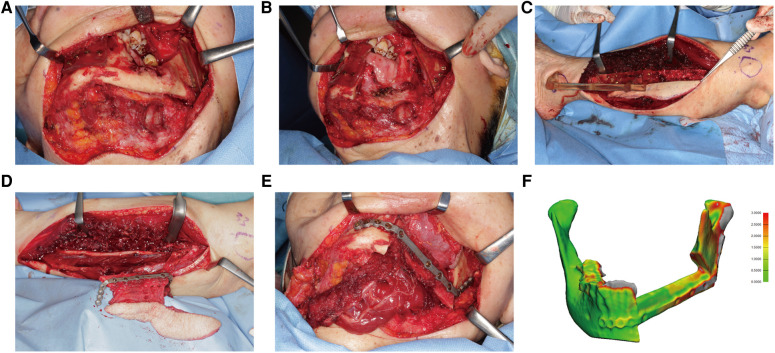
A 75-year-old female presented with osteoradionecrosis at the left mandible. (**A**) The mandible was exposed, and cutting guides were mounted to guide the segmental resection. Screw holes were drilled before osteotomy. (**B**) The mandible defect after resection was shown. (**C**) The fibula free flap was harvested with the cutting guide. (**D**) The fibula was fixed to the PSSP. (**E**) The “fibula-plate” was transferred to repair the mandibular defect. (**F**) The absolute distance deviation was 0.72 ± 0.75 mm.

Three representative cases of PSSPs-enhanced CAMR were depicted in Figures 6–8. All neo-mandibles exhibited a pleasing contour. The average absolute distance deviation of the mandible was 0.75 ± 0.20 mm. All patients recovered smoothly after surgery.

**Figure 9:**
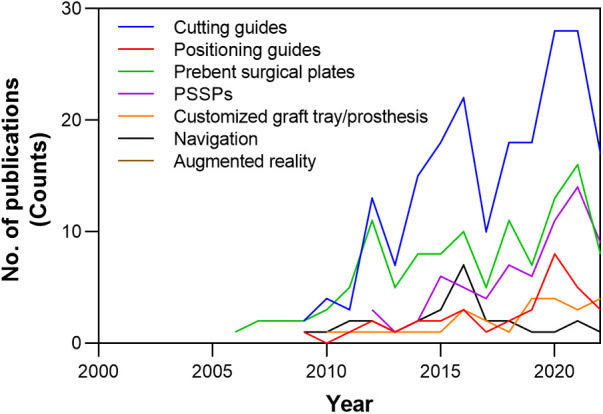
The evolution of hardware and devices used in CAMR, according to the number of academic publications over time in the literature.

The development of CAMR was characterized by a review of 8,672 articles (Supplementary Materials). After screening, a total of 266 studies were included for evaluation. The evolution of hardware and devices used in CAMR was plotted in Figure 9. The application of PSSPs increased and gradually caught up with conventional prebent surgical plates in academic publications. Four primary techniques of CAMR were summarized in Table 2. Compared to other techniques, PSSPs provided the benefits of matching structures and holes, contributing to the precise mandibular reconstruction.

**Table 2:** Major surgical techniques and devices of CAMR in literature.

No.	Hardware and devices					Comments
	Cutting guides	Positioning guides	Prebent surgical plates	PSSPs	Surgical navigation	
1	*✓*	*✓*				Rigid matching anatomical structures; no matching screw holes.
2	*✓*		*✓*			Matching anatomical structures; no matching screw holes.
3	*✓*			*✓*		Matching anatomical structures; matching screw holes; smooth procedures.
4					*✓*	Locating anatomical landmarks; tedious procedures; no hepatic feedbacks; mainly serving as an adjuvant technique.

## 4. Discussion

In this study, we confirmed that PSSPs could improve the precision of CAMR compared to prebent surgical plates. We demonstrated the clinical workflow of PSSPs and, for the first time, elucidated the benefits of matching bone structures and registering screw holes underlying precise surgery. Compared to other techniques, the cutting-edge PSSPs contributed to the enhanced precision of CAMR through the art of matching structures and holes.

Multiple measurements were used to assess the spatial deviations of CAMR in a comprehensive manner. Notably, the intercondylar length was used to evaluate bilateral condylar heads as a whole (6). According to relevant clinical studies, the deviation of intercondylar length was 2.6 ± 3.0 mm by PSSPs and 5.2 ± 4.2 mm by conventional surgical plates (6). Since adverse effects of soft tissue interference were avoided in our study, the spatial deviations were decreased in both groups. Similar results were found for the deviation of intergonial length. According to multiple indicators, PSSPs demonstrated a premium role in precise surgery. The main results were consistent with previous studies by other researchers (6, 7, 16–18).

**Figure 10:**
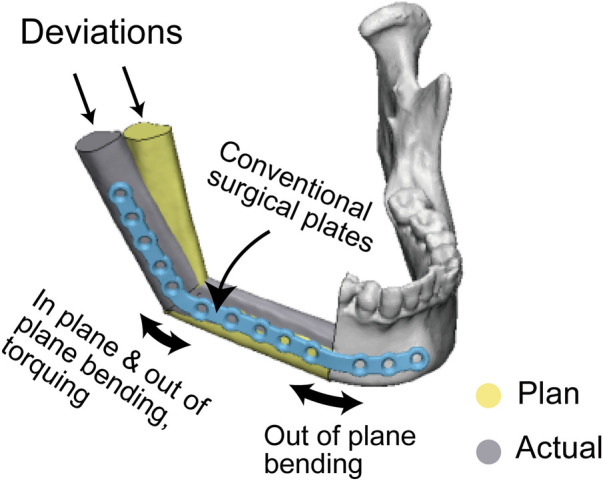
Manual plate bending and the source of reconstruction errors.

Interestingly, on the operated side, PSSPs demonstrated better results in the coronal and sagittal mandibular angles but not in the axial mandibular angle, which was explained by the complex plate bending at the mandibular angle. There were three types of bends to adapt a conventional surgical plate in the mandibular angle: in-plane bending, out-of-plane bending, and torqueing (Figure 10). The complex bending would induce more errors, signifying the benefits of PSSPs in restoring the mandibular angle. Ren et al. compared prebent plates with freehand surgery and found similar results to us. In their cases, the deviations of sagittal mandibular angle were 3.85 ± 1.68° and 5.88 ± 2.12°, respectively (19). On the contrary, the axial mandibular angle was mainly affected by the angular transition in the mental tubercle. Only out-of-plane bending was engaged in this region, and thus prebent surgical plates had comparable results as PSSPs.

The art of matching structures and holes in PSSPs-enhanced CAMR was confirmed by the submillimeter bone-plate distance and screw hole deviation. The optimal congruence between PSSPs and bone structures has been acknowledged in multiple studies. Ciocca et al. reported their pioneering application of PSSPs in mandibular reconstruction, which reproduced the anatomical contour ideally and provided surgeons with better procedural control (20). Wilde et al. used milled PSSPs for reconstruction, and all plates had a good fitness with bone (21). Yang et al. employed PSSPs to guide the folding and fixing of fibular segments, which resulted in streamlined surgery and enhanced precision (5, 6). Ong et al. used hydroformed PSSPs with better fitness than prebent plates (22). The geometric mismatch of prebent plates would drag fibular segments away from the accurate position and create deviations. Smith et al. declared that PSSPs had a more precise contour than prebent plates (23). Seier et al. also detected better bone contact using PSSPs and advocated PSSPs as a paradigm change in mandibular reconstruction (18). Zavattero et al. measured the accuracy of PSSPs one month after surgery and found that 80% of plates had a deviation within 2 mm (18). Kraeima et al. proposed biomechanically optimized PSSPs with an enlarged contact with underlying bone structures (3). In summary, the benefits of PSSPs have been widely accepted, and the art of matching structures and holes is revolutionizing the paradigm of mandibular reconstruction.

A review of CAMR also distinguished the benefits of PSSPs. The evolution of CAMR could be identified through the development and application of patient-specific devices (Supplementary Materials). The most used hardware and devices were cutting guides, positioning guides, prebent plates, PSSPs, and surgical navigation. Cutting guides were of multipurpose and designed for mandibular resection, bone graft osteotomy, soft tissue harvesting (24), screw hole drilling, and dental implant placement (8). Positioning guides directed the accurate position of bone grafts, mandibular stumps, and the reconstructed mandible. Prebent plates were manually bent according to rapid-prototyped models of the original mandible or virtually designed mandible. PSSPs were manufactured by metal casting, Computerized Numerical Control (CNC) milling, and additive manufacturing. PSSPs could be in the form of reconstruction plates, two-layer plates (9), miniplates (25), and temporomandibular joint (TMJ) prostheses (20). Surgical navigation was mainly adjunctive for cutting bone, drilling holes, and locating anatomical landmarks. Different combinations of devices were reported for CAMR, and four primary types were summarized below.

In type 1, cutting guides and positioning guides were used. Positioning guides provided rigid matching structures for the optimal arrangement of bone segments. Zheng et al. introduced accurate mandibular reconstruction using cutting and positioning guides (26). Because of the low cost and excellent accuracy, positioning guides were highly recommended in centers without PSSPs, which could be combined with prebent plates. However, surgical deviations resulting from plate bending could not be overlooked. There is no knowledge of whether PSSPs provided better or equivalent precision than positioning guides.

In type 2, cutting guides were combined with prebent surgical plates. Conventional off-the-shelf surgical plates were manually bended according to rapid-prototyped mandible models. Some studies confirmed that prebent plates simplified mandibular reconstruction and improved surgical outcomes compared to freehand surgery (27, 28). However, the lack of registered screw holes could unavoidably compromise the precision of surgery, as verified in the present study. In some pilot studies, prebent plates could be further combined with the “transfer key” or “predrilled cutting guides” to determine the relative position between prebent plates and bone, enhancing reconstruction precision (29–31). However, the additional procedures are time-consuming and not straightforward.

In type 3, PSSPs represented the cutting-edge technique of CAMR. PSSPs streamlined surgical procedures and enhanced surgical outcomes by the art of matching structures and holes, as demonstrated in the present study. Clinical evidence on benefits of PSSPs was accumulating. Lee et al. studied a cohort of 55 patients using PSSPs and found PSSPs contributed to fewer complications, reduced operative time, and shorter hospital stays (7). Zavattero et al. analyzed 54 patients with PSSPs and found the reconstruction accuracy within 3 mm (16). Yang et al. discovered that PSSPs reduced spatial deviations of TMJ after oncological mandibular reconstruction (32). To sum up, the benefits of PSSPs in CAMR would likely promote this new technology in the state-of-the-art clinical workflow of digital dentistry.

In type 4, surgical navigation was used alone or with other devices. Surgical navigation required intraoperative registration to locate anatomical landmarks, which might fit into the scope of matching structures, but without rigid registration and hepatic feedback. Shan et al. used surgical navigation for mandibular reconstruction (33). More than 90% of patients achieved an accuracy within 3 mm at one week after surgery. Yu et al. focused on secondary mandibular reconstruction and the reconstruction deviation was within 5 mm (34). Generally, surgical navigation was more accurate than freehand surgery. While in most circumstances, surgical navigation routinely served as an adjuvant technique combining other devices (35).

Above all, the premium role of PSSPs was distinguished by the benefits of matching structures and holes. Limitations of model surgery restricted the interpretation of quantitative results for clinical practice. The sample size was also small. However, the objectives of this article were fulfilled. The premium role of PSSPs in precise surgery was characterized, and their working principles in CAMR were demonstrated. A better understanding of the working principles would help reduce unexpected clinical problems in using PSSPs (36). The benefits of PSSPs would be more advocated in future.

## 5. Conclusions

The PSSPs-enhanced surgical precision was verified through the model surgery. The clinical workflow of PSSPs-enhanced CAMR was also demonstrated in human surgery. Compared to other techniques of CAMR, the cutting-edge PSSPs contributed to precise surgery through the art of matching structures and holes.

## Data Availability

The original contributions presented in the study are included in the article/**Supplementary Material**, further inquiries can be directed to the corresponding author/s.
