# Supplementary material for: Cutting-edge patient-specific surgical plates for computer-assisted mandibular reconstruction: The art of matching structures and holes in precise surgery
Source: Front Surg. 2023 Mar 9;10:1132669. doi: 10.3389/fsurg.2023.1132669 (PMC10033664; doi:10.3389/fsurg.2023.1132669)
Supplement: Supplementary file 1 [file Datasheet1.docx]

Supplementary Material

Cutting-Edge Patient-Specific Surgical Plates for Computer-Assisted Mandibular Reconstruction: The Art of Matching Structures and Holes in Precise Surgery

Renshun Liu, Yuxiong Su, Weifa Yang*, Jingya Pu, Chunyu Zhang

*** Correspondence:** Weifa Yang: [yangweifa@gmail.com](mailto:yangweifa@gmail.com)

# Supplementary Figures and Tables

## Supplementary Figures


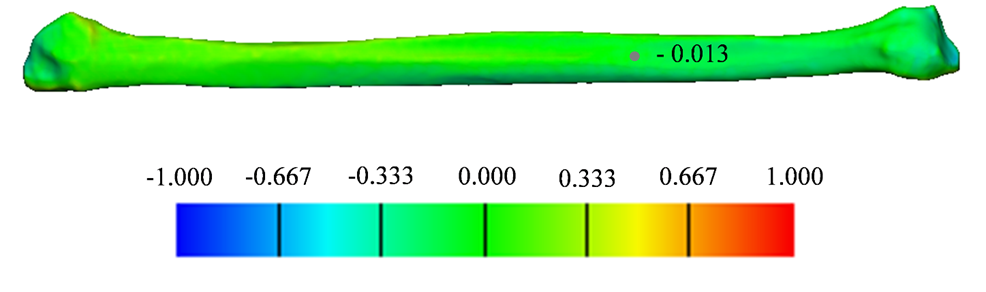


**Supplementary Figure 1.** The accuracy and reliability of rapid prototyped models. The digitized fibula was superimposed on the virtual fibula to verify the accuracy of rapid prototyping. The absolute distance deviation was -0.013 ± 0.186 mm.

**
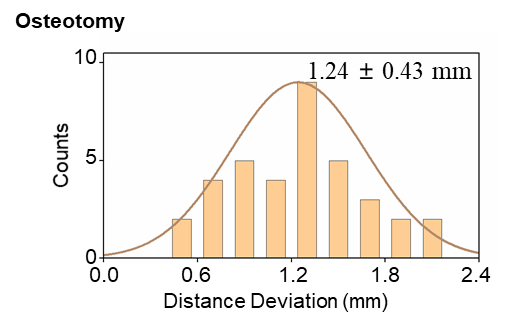
**

**Supplementary Figure 2.** The distance deviation of osteotomy assisted by cutting guides.


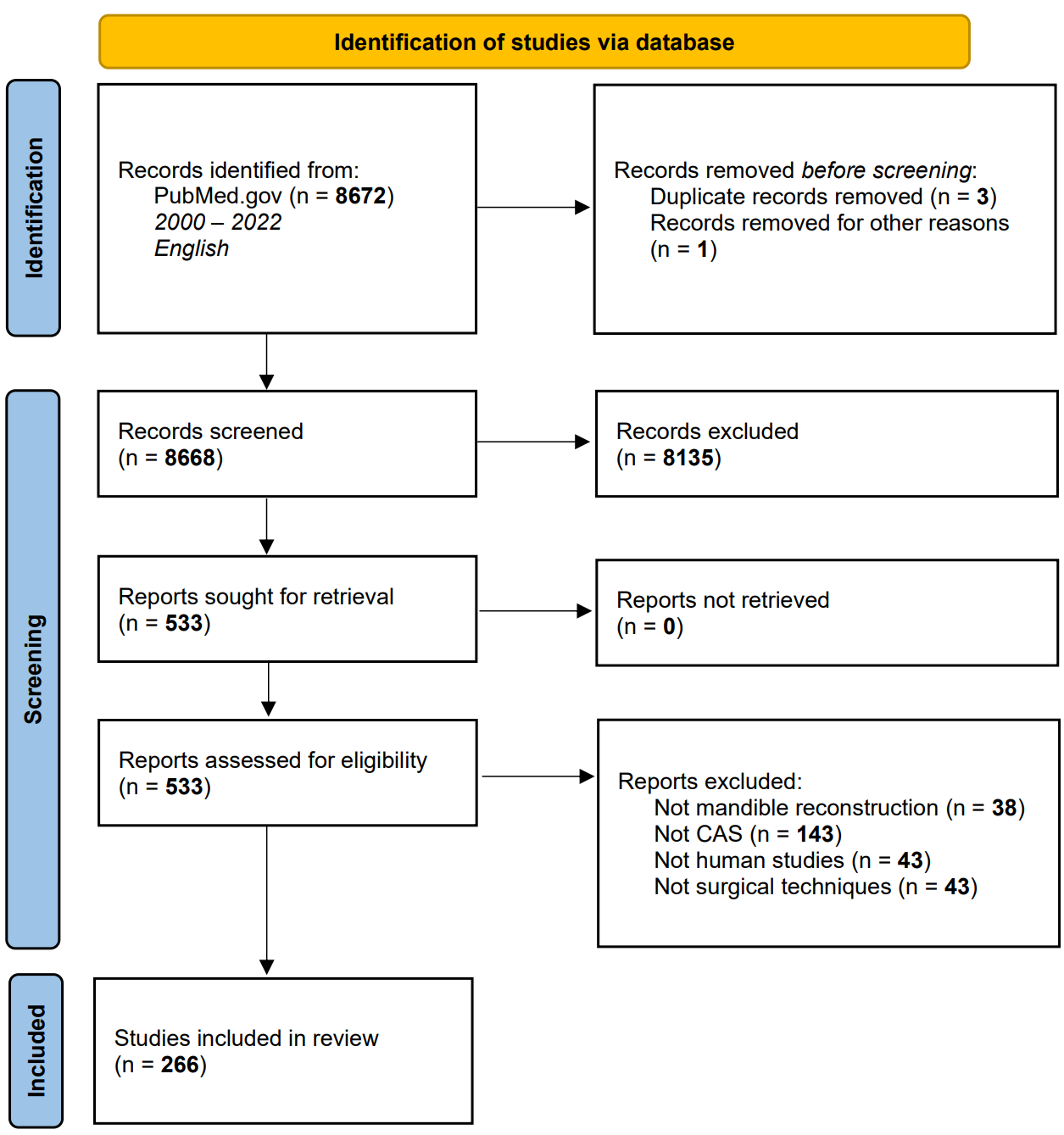


**Supplementary Figure 3.** PRISMA 2020 flow diagram for the systematic review of CAMR techniques in literature.

## Supplementary Tables

**Supplementary Table 1.** Literature search strategy in PubMed.gov.

| **Included domains** | **Excluded domains** | **Codes** | **Keywords/Filters** |
| --- | --- | --- | --- |
| Mandible |  | #1 | mandible |
|  |  | #2 | mandibular |
| Reconstruction |  | #3 | reconstruction |
|  |  | #4 | reconstructive |
|  | Trauma | #5 | fracture |
| Year |  | #6 | 2000:2022[pdat] |
| Language |  | #7 | English |
|  |  | #8 | #1 OR #2 |
|  |  | #9 | #3 OR #4 |
| Search: **#8 AND #9 NOT #5 AND #6**  Filters: **English** | | | |
| **Query:**  ((("mandible"[MeSH Terms] OR "mandible"[All Fields] OR "mandibles"[All Fields] OR "mandible s"[All Fields] OR ("mandible"[MeSH Terms] OR "mandible"[All Fields] OR "mandibular"[All Fields] OR "mandibulars"[All Fields])) AND ("reconstruct"[All Fields] OR "reconstructability"[All Fields] OR "reconstructable"[All Fields] OR "reconstructed"[All Fields] OR "reconstructible"[All Fields] OR "reconstructing"[All Fields] OR "reconstructional"[All Fields] OR "reconstructive surgical procedures"[MeSH Terms] OR ("reconstructive"[All Fields] AND "surgical"[All Fields] AND "procedures"[All Fields]) OR "reconstructive surgical procedures"[All Fields] OR "reconstruction"[All Fields] OR "reconstructions"[All Fields] OR "reconstructive"[All Fields] OR "reconstructs"[All Fields] OR ("reconstruct"[All Fields] OR "reconstructability"[All Fields] OR "reconstructable"[All Fields] OR "reconstructed"[All Fields] OR "reconstructible"[All Fields] OR "reconstructing"[All Fields] OR "reconstructional"[All Fields] OR "reconstructive surgical procedures"[MeSH Terms] OR ("reconstructive"[All Fields] AND "surgical"[All Fields] AND "procedures"[All Fields]) OR "reconstructive surgical procedures"[All Fields] OR "reconstruction"[All Fields] OR "reconstructions"[All Fields] OR "reconstructive"[All Fields] OR "reconstructs"[All Fields]))) NOT ("fractur"[All Fields] OR "fractural"[All Fields] OR "fracture s"[All Fields] OR "fractures, bone"[MeSH Terms] OR ("fractures"[All Fields] AND "bone"[All Fields]) OR "bone fractures"[All Fields] OR "fracture"[All Fields] OR "fractured"[All Fields] OR "fractures"[All Fields] OR "fracturing"[All Fields])) AND ((english[Filter]) AND (2000:2023[pdat])) | | | |

**Supplementary Table 2.** A summary of CAMR techniques in literature (included in review) in alphabetical order.

| **Techniques** | **Articles** | **Counts** |
| --- | --- | --- |
| **Augmented reality + cutting guides** | | **1** |
|  | [1] S. Battaglia, G. Badiali, L. Cercenelli, B. Bortolani, E. Marcelli, R. Cipriani, F. Contedini, C. Marchetti, A. Tarsitano, Combination of CAD/CAM and Augmented Reality in Free Fibula Bone Harvest, Plast Reconstr Surg Glob Open 7(11) (2019) e2510. |  |
| **Customized graft tray/prosthesis** | | **22** |
|  | [1] L.B. Zhou, H.T. Shang, L.S. He, B. Bo, G.C. Liu, Y.P. Liu, J.L. Zhao, Accurate reconstruction of discontinuous mandible using a reverse engineering/computer-aided design/rapid prototyping technique: a preliminary clinical study, J Oral Maxillofac Surg 68(9) (2010) 2115-21. |  |
|  | [2] J.S. Hou, M. Chen, C.B. Pan, Q. Tao, J.G. Wang, C. Wang, B. Zhang, H.Z. Huang, Immediate reconstruction of bilateral mandible defects: management based on computer-aided design/computer-aided manufacturing rapid prototyping technology in combination with vascularized fibular osteomyocutaneous flap, J Oral Maxillofac Surg 69(6) (2011) 1792-7. |  |
|  | [3] J.S. Hou, M. Chen, C.B. Pan, M. Wang, J.G. Wang, B. Zhang, Q. Tao, C. Wang, H.Z. Huang, Application of CAD/CAM-assisted technique with surgical treatment in reconstruction of the mandible, J Craniomaxillofac Surg 40(8) (2012) e432-7. |  |
|  | [4] N. Fernandes, J. van den Heever, K. Hoek, G. Booysen, Customized reconstruction of an extensive mandibular defect: A clinical report, J Prosthet Dent 116(6) (2016) 928-931. |  |
|  | [5] T. Ikawa, Y. Shigeta, R. Hirabayashi, S. Hirai, K. Hirai, N. Harada, N. Kawamura, T. Ogawa, Computer assisted mandibular reconstruction using a custom-made titan mesh tray and removable denture based on the top-down treatment technique, J Prosthodont Res 60(4) (2016) 321-331. |  |
|  | [6] A. Ow, W. Tan, L. Pienkowski, Mandibular Reconstruction Using a Custom-Made Titanium Prosthesis: A Case Report on the Use of Virtual Surgical Planning and Computer-Aided Design/Computer-Aided Manufacturing, Craniomaxillofac Trauma Reconstr 9(3) (2016) 246-50. |  |
|  | [7] Y.W. Lee, H.J. You, J.A. Jung, D.W. Kim, Mandibular reconstruction using customized three-dimensional titanium implant, Arch Craniofac Surg 19(2) (2018) 152-156. |  |
|  | [8] D. Öhman, C. Schaefer, U. Nannmark, G. Kjeller, J. Malmström, Mandible reconstruction with patient-specific implants: Case report of five consecutive patients, Int J Oral Maxillofac Implants 34(1) (2019) e7–e11. |  |
|  | [9] V. U, D. Mehrotra, D. Howlader, P.K. Singh, S. Gupta, Patient Specific Three-Dimensional Implant for Reconstruction of Complex Mandibular Defect, J Craniofac Surg 30(4) (2019) e308-e311. |  |
|  | [10] J. Zheng, X. Chen, W. Jiang, S. Zhang, M. Chen, C. Yang, An innovative total temporomandibular joint prosthesis with customized design and 3D printing additive fabrication: a prospective clinical study, J Transl Med 17(1) (2019) 4. |  |
|  | [11] J.S. Zheng, X.H. Liu, X.Z. Chen, W.B. Jiang, A. Abdelrehem, S.Y. Zhang, M.J. Chen, C. Yang, Customized skull base-temporomandibular joint combined prosthesis with 3D-printing fabrication for craniomaxillofacial reconstruction: a preliminary study, Int J Oral Maxillofac Surg 48(11) (2019) 1440-1447. |  |
|  | [12] F. Grecchi, P.A. Zecca, A. Macchi, A. Mangano, F. Riva, E. Grecchi, C. Mangano, Full-Digital Workflow for Fabricating a Custom-Made Direct Metal Laser Sintering (DMLS) Mandibular Implant: A Case Report, Int J Environ Res Public Health 17(8) (2020). |  |
|  | [13] F. Ricotta, S. Battaglia, F. Bolognesi, F. Ceccariglia, C. Marchetti, A. Tarsitano, Use of CAD-CAM Bridging Mandibular Prosthesis in Osteonecrosis of the Jaw: The Experience of Our School, J Clin Med 9(11) (2020). |  |
|  | [14] Y. Xia, Z.C. Feng, C. Li, H. Wu, C. Tang, L. Wang, H. Li, Application of additive manufacturing in customized titanium mandibular implants for patients with oral tumors, Oncol Lett 20(4) (2020) 51. |  |
|  | [15] J.S. Zheng, X.H. Liu, A. Ahmed, M.J. Chen, S.Y. Zhang, C. Yang, Endoscopically assisted fixation of the custom-made total temporomandibular joint prosthesis in TMJ Yang's system through a modified preauricular approach, Int J Oral Maxillofac Surg 49(2) (2020) 224-229. |  |
|  | [16] D.M. Chernohorskyi, Y.V. Chepurnyi, O.A. Kanyura, A.V. Kopchak, TOTAL MANDIBULAR DEFECT RECONSTRUCTION BY TOTAL TITANIUM PATIENT-SPECIFIC IMPLANT: CLINICAL EFFICACY AND LONG TERM FOLLOW UP. CLINICAL CASE, Wiad Lek 74(4) (2021) 1037-1041. |  |
|  | [17] K. Darwich, M.B. Ismail, M.Y.A. Al-Mozaiek, A. Alhelwani, Reconstruction of mandible using a computer-designed 3D-printed patient-specific titanium implant: a case report, Oral Maxillofac Surg 25(1) (2021) 103-111. |  |
|  | [18] A. Tarsitano, S. Battaglia, G. Corinaldesi, C. Marchetti, G. Pellegrino, L. Ciocca, Mandibular reconstruction using a new design for a patient-specific plate to support a fibular free flap and avoid double-barrel technique, Acta Otorhinolaryngol Ital 41(3) (2021) 230-235. |  |
|  | [19] C.M. Ardila, Y. Hernández-Arenas, E. Álvarez-Martínez, Mandibular Body Reconstruction Utilizing a Three-Dimensional Custom-Made Porous Titanium Plate: A Four-Year Follow-Up Clinical Report, Case Rep Dent 2022 (2022) 5702066. |  |
|  | [20] A.P. de Sousa Gil, B.D. Velasques, N. Uzun, O.L. Haas, R.B. de Oliveira, Total Customized Alloplastic Reconstruction for Treatment of Severe Temporomandibular Joint Pathologic Conditions: A Case Series of Combined Intraoral and Extraoral Approach, J Craniofac Surg 33(3) (2022) e250-e253. |  |
|  | [21] H.K. Lim, Y.J. Choi, W.C. Choi, I.S. Song, U.L. Lee, Reconstruction of maxillofacial bone defects using patient-specific long-lasting titanium implants, Sci Rep 12(1) (2022) 7538. |  |
|  | [22] J.S. Zheng, Z.X. Jiao, X. Wei, M.J. Chen, A. Ahmed, C. Yang, Accuracy of digital templates for guidance of custom-made total temporomandibular joint replacement, Int J Oral Maxillofac Surg (2022). |  |
| **Cutting guides (only)** | | **22** |
|  | [1] C. Leiggener, E. Messo, A. Thor, H.F. Zeilhofer, J.M. Hirsch, A selective laser sintering guide for transferring a virtual plan to real time surgery in composite mandibular reconstruction with free fibula osseous flaps, Int J Oral Maxillofac Surg 38(2) (2009) 187-92. |  |
|  | [2] Y. Yamanaka, H. Yajima, T. Kirita, H. Shimomura, S. Tamaki, K. Aoki, N. Yamakawa, Y. Imai, Mandibular reconstruction with vascularised fibular osteocutaneous flaps using prefabricated stereolithographic mandibular model, J Plast Reconstr Aesthet Surg 63(10) (2010) 1751-3. |  |
|  | [3] A. Modabber, M. Gerressen, M.B. Stiller, N. Noroozi, A. Füglein, F. Hölzle, D. Riediger, A. Ghassemi, Computer-assisted mandibular reconstruction with vascularized iliac crest bone graft, Aesthetic Plast Surg 36(3) (2012) 653-9. |  |
|  | [4] J.P. Levine, J.S. Bae, M. Soares, L.E. Brecht, P.B. Saadeh, D.J. Ceradini, D.L. Hirsch, Jaw in a day: total maxillofacial reconstruction using digital technology, Plast Reconstr Surg 131(6) (2013) 1386-1391. |  |
|  | [5] C.V. Thomas, K.G. McMillan, P. Jeynes, T. Martin, S. Parmar, Use of a titanium cutting guide to assist raising the composite radial forearm free flap, Int J Oral Maxillofac Surg 42(11) (2013) 1414-7. |  |
|  | [6] W.S. Jeong, J.W. Choi, S.H. Choi, Computer simulation surgery for mandibular reconstruction using a fibular osteotomy guide, Arch Plast Surg 41(5) (2014) 584-7. |  |
|  | [7] A. Modabber, N. Ayoub, S.C. Möhlhenrich, E. Goloborodko, T.T. Sönmez, M. Ghassemi, C. Loberg, B. Lethaus, A. Ghassemi, F. Hölzle, The accuracy of computer-assisted primary mandibular reconstruction with vascularized bone flaps: iliac crest bone flap versus osteomyocutaneous fibula flap, Med Devices (Auckl) 7 (2014) 211-7. |  |
|  | [8] J. Rustemeyer, A. Busch, A. Sari-Rieger, Application of computer-aided designed/computer-aided manufactured techniques in reconstructing maxillofacial bony structures, Oral Maxillofac Surg 18(4) (2014) 471-6. |  |
|  | [9] J. Kraeima, R.H. Schepers, P.M. van Ooijen, R.J. Steenbakkers, J.L. Roodenburg, M.J. Witjes, Integration of oncologic margins in three-dimensional virtual planning for head and neck surgery, including a validation of the software pathway, J Craniomaxillofac Surg 43(8) (2015) 1374-9. |  |
|  | [10] A. Modabber, S.C. Möhlhenrich, N. Ayoub, M. Hajji, S. Raith, S. Reich, T. Steiner, A. Ghassemi, F. Hölzle, Computer-Aided Mandibular Reconstruction With Vascularized Iliac Crest Bone Flap and Simultaneous Implant Surgery, J Oral Implantol 41(5) (2015) e189-94. |  |
|  | [11] P. Olsson, F. Nysjö, A. Rodríguez-Lorenzo, A. Thor, J.M. Hirsch, I.B. Carlbom, Haptics-assisted Virtual Planning of Bone, Soft Tissue, and Vessels in Fibula Osteocutaneous Free Flaps, Plast Reconstr Surg Glob Open 3(8) (2015) e479. |  |
|  | [12] D. Culié, O. Dassonville, G. Poissonnet, J.C. Riss, J. Fernandez, A. Bozec, Virtual planning and guided surgery in fibular free-flap mandibular reconstruction: A 29-case series, Eur Ann Otorhinolaryngol Head Neck Dis 133(3) (2016) 175-8. |  |
|  | [13] T. Numajiri, H. Nakamura, Y. Sowa, K. Nishino, Low-cost Design and Manufacturing of Surgical Guides for Mandibular Reconstruction Using a Fibula, Plast Reconstr Surg Glob Open 4(7) (2016) e805. |  |
|  | [14] W.L. Weijs, C. Coppen, R. Schreurs, R.D. Vreeken, A.C. Verhulst, M.A. Merkx, S.J. Bergé, T.J. Maal, Accuracy of virtually 3D planned resection templates in mandibular reconstruction, J Craniomaxillofac Surg 44(11) (2016) 1828-1832. |  |
|  | [15] J. Weitz, F.J. Bauer, A. Hapfelmeier, N.H. Rohleder, K.D. Wolff, M.R. Kesting, Accuracy of mandibular reconstruction by three-dimensional guided vascularised fibular free flap after segmental mandibulectomy, Br J Oral Maxillofac Surg 54(5) (2016) 506-10. |  |
|  | [16] O. Emodi, D. Shilo, Y. Israel, A. Rachmiel, Three-dimensional planning and printing of guides and templates for reconstruction of the mandibular ramus and condyle using autogenous costochondral grafts, Br J Oral Maxillofac Surg 55(1) (2017) 102-104. |  |
|  | [17] X. Xu, H. Ma, S. Jin, One-Stage Treatment of Giant Condylar Osteoma: Alloplastic Total Temporomandibular Joint Replacement Aided by Digital Templates, J Craniofac Surg 29(3) (2018) 636-639. |  |
|  | [18] V. Saini, S. Gaba, S. Sharma, P. Kalra, R.K. Sharma, Assessing the Role of Virtual Surgical Planning in Mandibular Reconstruction With Free Fibula Osteocutaneous Graft, J Craniofac Surg 30(6) (2019) e563-e566. |  |
|  | [19] R.N. Solis, J. Mahaney, R. Mohhebali, S. Laks, M.K. Wax, D. Petrisor, H.C. Brockhoff, 2nd, Digital imaging evaluation of the scapula for prediction of endosteal implant placement in reconstruction of oromandibular defects with scapular free flaps, Microsurgery 39(8) (2019) 730-736. |  |
|  | [20] J.T. Stranix, C.S. Stern, M. Rensberger, I. Ganly, J.O. Boyle, R.J. Allen, Jr., J.J. Disa, B.J. Mehrara, E.S. Garfein, E. Matros, A Virtual Surgical Planning Algorithm for Delayed Maxillomandibular Reconstruction, Plast Reconstr Surg 143(4) (2019) 1197-1206. |  |
|  | [21] C. Druelle, M. Schlund, J.C. Lutz, M. Constant, G. Raoul, R. Nicot, A modified method for a customized harvest of fibula free flap in maxillofacial reconstruction, J Stomatol Oral Maxillofac Surg 121(1) (2020) 74-76. |  |
|  | [22] A. Piotrowska-Seweryn, C. Szymczyk, D.A. Walczak, Ł. Krakowczyk, A. Maciejewski, G. Hadasik, J. Wierzgoń, R. Szumniak, P. Drozdowski, P. Paul, M. Grajek, Fibular Free Flap and Iliac Crest Free Flap Mandibular Reconstruction In Patients With Mandibular Ameloblastomas, J Craniofac Surg (2022). |  |
| **Cutting guides + patient-specific implant bar** | | **4** |
|  | [1] R.H. Schepers, G.M. Raghoebar, A. Vissink, L.U. Lahoda, W.J. Van der Meer, J.L. Roodenburg, H. Reintsema, M.J. Witjes, Fully 3-dimensional digitally planned reconstruction of a mandible with a free vascularized fibula and immediate placement of an implant-supported prosthetic construction, Head Neck 35(4) (2013) E109-14. |  |
|  | [2] C. Freudlsperger, J.P. Bodem, E. Engel, J. Hoffmann, Mandibular reconstruction with a prefabricated free vascularized fibula and implant-supported prosthesis based on fully three-dimensional virtual planning, J Craniofac Surg 25(3) (2014) 980-2. |  |
|  | [3] F.P. Koch, E. Götze, V.V. Kumar, P. Schulz, S. Wentaschek, W. Wagner, A bar-retained overdenture as an external fixator device in a three-dimensional CAD/CAM-based surgical reconstruction of the mandible, J Craniomaxillofac Surg 43(8) (2015) 1447-51. |  |
|  | [4] R.H. Schepers, J. Kraeima, A. Vissink, L.U. Lahoda, J.L. Roodenburg, H. Reintsema, G.M. Raghoebar, M.J. Witjes, Accuracy of secondary maxillofacial reconstruction with prefabricated fibula grafts using 3D planning and guided reconstruction, J Craniomaxillofac Surg 44(4) (2016) 392-9. |  |
| **Cutting guides + positioning guides** | | **11** |
|  | [1] D.L. Shu, X.Z. Liu, B. Guo, W. Ran, X. Liao, Y.Y. Zhang, Accuracy of using computer-aided rapid prototyping templates for mandible reconstruction with an iliac crest graft, World J Surg Oncol 12 (2014) 190. |  |
|  | [2] J.W. Lee, B.J. Choi, D.W. Lee, Y.D. Kwon, Double-barrelled vascularised fibular free flap using computer-assisted preoperative planning and a surgical template for accurate reconstruction of a segmental mandibular defect, Br J Oral Maxillofac Surg 54(1) (2016) 102-3. |  |
|  | [3] S.H. Lim, M.K. Kim, S.H. Kang, Precision of fibula positioning guide in mandibular reconstruction with a fibula graft, Head Face Med 12 (2016) 7. |  |
|  | [4] L. Zhang, Z. Liu, B. Li, H. Yu, S.G. Shen, X. Wang, Evaluation of computer-assisted mandibular reconstruction with vascularized fibular flap compared to conventional surgery, Oral Surg Oral Med Oral Pathol Oral Radiol 121(2) (2016) 139-48. |  |
|  | [5] Y. Ren, Q. Xi, L. Zhang, H. Liu, Y. Shi, M. Zhang, Computer-Aided Design and Three-Dimensional-Printed Surgical Templates for Second-Stage Mandibular Reconstruction, J Craniofac Surg 29(8) (2018) 2101-2105. |  |
|  | [6] T.F. Wu, J.Y. Liu, Y.S. Li, B. Liu, Matching locating holes in multiple plates to record bone position for accurate reconstruction after segmental mandibulectomy, Int J Oral Maxillofac Surg 48(12) (2019) 1516-1519. |  |
|  | [7] S.R. Kim, S. Jang, K.M. Ahn, J.H. Lee, Evaluation of Effective Condyle Positioning Assisted by 3D Surgical Guide in Mandibular Reconstruction Using Osteocutaneous Free Flap, Materials (Basel) 13(10) (2020). |  |
|  | [8] H. Sun, J. Zhang, B. Li, Z. Liu, S. Shen, X. Wang, Accuracy of a new custom-made bone-supported osteotomy and repositioning guide system for reconstruction of the mandibular ramus using costochondral grafts: a preliminary study, Br J Oral Maxillofac Surg 58(1) (2020) 51-56. |  |
|  | [9] R. Yamochi, T. Numajiri, H. Nakamura, D. Morita, Y. Sowa, Innovative CAD/CAM Guide for Mandibular Reconstruction with Metallic Condylar Head and Free Fibular Flap, Plast Reconstr Surg Glob Open 8(9) (2020) e3088. |  |
|  | [10] T.Y. Huang, C.Y. Fang, K.C. Lin, Y. Ashikaga, Utilizing virtual surgical planning and three-dimensional-printed osteotomy guides in fibular free flap reconstruction can achieve a better result in mandibular osteoradionecrosis patient, J Dent Sci 17(1) (2022) 630-632. |  |
|  | [11] G. Xu, J. Jia, X. Xiong, L. Peng, L.L. Bu, X. Wang, Mandibular Reconstruction With the Contralateral Vascularized Iliac Flap Using Individual Design: Iliac Crest Used to Reconstruct the Ramus and the Anterior Border of the Iliac Wing Used to Reconstruct the Inferior Border: A Case Report, Front Surg 9 (2022) 924241. |  |
| **Cutting guides + prebent surgical plates** | | **65** |
|  | [1] S.M. Roser, S. Ramachandra, H. Blair, W. Grist, G.W. Carlson, A.M. Christensen, K.A. Weimer, M.B. Steed, The accuracy of virtual surgical planning in free fibula mandibular reconstruction: comparison of planned and final results, J Oral Maxillofac Surg 68(11) (2010) 2824-32. |  |
|  | [2] B. Sharaf, J.P. Levine, D.L. Hirsch, J.A. Bastidas, B.A. Schiff, E.S. Garfein, Importance of computer-aided design and manufacturing technology in the multidisciplinary approach to head and neck reconstruction, J Craniofac Surg 21(4) (2010) 1277-80. |  |
|  | [3] A.K. Antony, W.F. Chen, A. Kolokythas, K.A. Weimer, M.N. Cohen, Use of virtual surgery and stereolithography-guided osteotomy for mandibular reconstruction with the free fibula, Plast Reconstr Surg 128(5) (2011) 1080-1084. |  |
|  | [4] N.T. Haddock, C. Monaco, K.A. Weimer, D.L. Hirsch, J.P. Levine, P.B. Saadeh, Increasing bony contact and overlap with computer-designed offset cuts in free fibula mandible reconstruction, J Craniofac Surg 23(6) (2012) 1592-5. |  |
|  | [5] P. Infante-Cossio, P. Gacto-Sanchez, T. Gomez-Cia, G. Gomez-Ciriza, Stereolithographic cutting guide for fibula osteotomy, Oral Surg Oral Med Oral Pathol Oral Radiol 113(6) (2012) 712-3; author reply 712. |  |
|  | [6] A. Patel, J. Levine, L. Brecht, P. Saadeh, D.L. Hirsch, Digital technologies in mandibular pathology and reconstruction, Atlas Oral Maxillofac Surg Clin North Am 20(1) (2012) 95-106. |  |
|  | [7] Y. Shen, J. Sun, J. Li, T. Ji, M.M. Li, W. Huang, M. Hu, Using computer simulation and stereomodel for accurate mandibular reconstruction with vascularized iliac crest flap, Oral Surg Oral Med Oral Pathol Oral Radiol 114(2) (2012) 175-82. |  |
|  | [8] J. Sink, D. Hamlar, D. Kademani, S.S. Khariwala, Computer-aided stereolithography for presurgical planning in fibula free tissue reconstruction of the mandible, J Reconstr Microsurg 28(6) (2012) 395-403. |  |
|  | [9] R. Winters, A. Saad, D.D. Beahm, M.W. Wise, H. St Hilaire, Total autogenous mandibular reconstruction using virtual surgical planning, J Craniofac Surg 23(5) (2012) e405-7. |  |
|  | [10] E.J. Moore, M.L. Hinni, K. Arce, T. Salinas, Mandibular alveolar reconstruction using three-dimensional planning, Curr Opin Otolaryngol Head Neck Surg 21(4) (2013) 328-34. |  |
|  | [11] A. Saad, R. Winters, M.W. Wise, C.L. Dupin, H. St Hilaire, Virtual surgical planning in complex composite maxillofacial reconstruction, Plast Reconstr Surg 132(3) (2013) 626-633. |  |
|  | [12] T. Avraham, P. Franco, L.E. Brecht, D.J. Ceradini, P.B. Saadeh, D.L. Hirsch, J.P. Levine, Functional outcomes of virtually planned free fibula flap reconstruction of the mandible, Plast Reconstr Surg 134(4) (2014) 628e-634e. |  |
|  | [13] N. Ayoub, A. Ghassemi, M. Rana, M. Gerressen, D. Riediger, F. Hölzle, A. Modabber, Evaluation of computer-assisted mandibular reconstruction with vascularized iliac crest bone graft compared to conventional surgery: a randomized prospective clinical trial, Trials 15 (2014) 114. |  |
|  | [14] N.K. Kim, H.Y. Kim, H.J. Kim, I.H. Cha, W. Nam, Considerations and Protocols in Virtual Surgical Planning of Reconstructive Surgery for More Accurate and Esthetic Neomandible with Deep Circumflex Iliac Artery Free Flap, Maxillofac Plast Reconstr Surg 36(4) (2014) 161-7. |  |
|  | [15] E. Matros, C.R. Albornoz, M. Rensberger, K. Weimer, E.S. Garfein, Computer-assisted design and computer-assisted modeling technique optimization and advantages over traditional methods of osseous flap reconstruction, J Reconstr Microsurg 30(5) (2014) 289-96. |  |
|  | [16] P. Metzler, E.J. Geiger, A. Alcon, X. Ma, D.M. Steinbacher, Three-dimensional virtual surgery accuracy for free fibula mandibular reconstruction: planned versus actual results, J Oral Maxillofac Surg 72(12) (2014) 2601-12. |  |
|  | [17] K.A. Rodby, S. Turin, R.J. Jacobs, J.F. Cruz, V.J. Hassid, A. Kolokythas, A.K. Antony, Advances in oncologic head and neck reconstruction: systematic review and future considerations of virtual surgical planning and computer aided design/computer aided modeling, J Plast Reconstr Aesthet Surg 67(9) (2014) 1171-85. |  |
|  | [18] R. Villar-Puchades, B. Ramos-Medina, Virtual surgical planning for extensive fibrous dysplasia in the mandible, Aesthetic Plast Surg 38(5) (2014) 941-5. |  |
|  | [19] H. Hanken, C. Schablowsky, R. Smeets, M. Heiland, S. Sehner, B. Riecke, I. Nourwali, O. Vorwig, A. Gröbe, A. Al-Dam, Virtual planning of complex head and neck reconstruction results in satisfactory match between real outcomes and virtual models, Clin Oral Investig 19(3) (2015) 647-56. |  |
|  | [20] J. Rustemeyer, A. Sari-Rieger, A. Melenberg, A. Busch, Comparison of intraoperative time measurements between osseous reconstructions with free fibula flaps applying computer-aided designed/computer-aided manufactured and conventional techniques, Oral Maxillofac Surg 19(3) (2015) 293-300. |  |
|  | [21] R. Sieira Gil, A.M. Roig, C.A. Obispo, A. Morla, C.M. Pagès, J.L. Perez, Surgical planning and microvascular reconstruction of the mandible with a fibular flap using computer-aided design, rapid prototype modelling, and precontoured titanium reconstruction plates: a prospective study, Br J Oral Maxillofac Surg 53(1) (2015) 49-53. |  |
|  | [22] E. Stirling Craig, M. Yuhasz, A. Shah, J. Blumberg, J. Salomon, R. Lowlicht, S. Fusi, D.M. Steinbacher, Simulated surgery and cutting guides enhance spatial positioning in free fibular mandibular reconstruction, Microsurgery 35(1) (2015) 29-33. |  |
|  | [23] G. Succo, M. Berrone, B. Battiston, P. Tos, F. Goia, P. Appendino, E. Crosetti, Step-by-step surgical technique for mandibular reconstruction with fibular free flap: application of digital technology in virtual surgical planning, Eur Arch Otorhinolaryngol 272(6) (2015) 1491-501. |  |
|  | [24] J.M. Toto, E.I. Chang, R. Agag, K. Devarajan, S.A. Patel, N.S. Topham, Improved operative efficiency of free fibula flap mandible reconstruction with patient-specific, computer-guided preoperative planning, Head Neck 37(11) (2015) 1660-4. |  |
|  | [25] E. Zavattero, P. Garzino-Demo, M. Fasolis, G. Ramieri, To computer-aided design and manufacturing or not to computer-aided design and manufacturing? Free fibula flap with computer-aided technique for mandibular reconstruction, J Craniofac Surg 26(3) (2015) e206-9. |  |
|  | [26] E.I. Chang, Long-Term Operative Outcomes of Preoperative Computed Tomography-Guided Virtual Surgical Planning for Osteocutaneous Free Flap Mandible Reconstruction, Plast Reconstr Surg 138(3) (2016) 559e-560e. |  |
|  | [27] M. Kääriäinen, M. Kuuskeri, G. Gremoutis, H. Kuokkanen, A. Miettinen, J. Laranne, Utilization of Three-Dimensional Computer-Aided Preoperative Virtual Planning and Manufacturing in Maxillary and Mandibular Reconstruction with a Microvascular Fibula Flap, J Reconstr Microsurg 32(2) (2016) 137-41. |  |
|  | [28] J.Y. Kim, W.S. Kim, E.C. Choi, W. Nam, The Role of Virtual Surgical Planning in the Era of Robotic Surgery, Yonsei Med J 57(1) (2016) 265-8. |  |
|  | [29] G. Kokosis, R. Schmitz, D.B. Powers, D. Erdmann, Mandibular Reconstruction Using the Free Vascularized Fibula Graft: An Overview of Different Modifications, Arch Plast Surg 43(1) (2016) 3-9. |  |
|  | [30] M. Mottini, S.M. Seyed Jafari, M. Shafighi, B. Schaller, New approach for virtual surgical planning and mandibular reconstruction using a fibula free flap, Oral Oncol 59 (2016) e6-e9. |  |
|  | [31] Y.Y. Wang, H.Q. Zhang, S. Fan, D.M. Zhang, Z.Q. Huang, W.L. Chen, J.T. Ye, J.S. Li, Mandibular reconstruction with the vascularized fibula flap: comparison of virtual planning surgery and conventional surgery, Int J Oral Maxillofac Surg 45(11) (2016) 1400-1405. |  |
|  | [32] X. Yuan, M. Xuan, W. Tian, J. Long, Application of digital surgical guides in mandibular resection and reconstruction with fibula flaps, Int J Oral Maxillofac Surg 45(11) (2016) 1406-1409. |  |
|  | [33] R. Bosc, B. Hersant, R. Carloni, J. Niddam, J. Bouhassira, H. De Kermadec, E. Bequignon, T. Wojcik, M. Julieron, J.P. Meningaud, Mandibular reconstruction after cancer: an in-house approach to manufacturing cutting guides, Int J Oral Maxillofac Surg 46(1) (2017) 24-31. |  |
|  | [34] L. Ganry, J. Quilichini, C.M. Bandini, P. Leyder, B. Hersant, J.P. Meningaud, Three-dimensional surgical modelling with an open-source software protocol: study of precision and reproducibility in mandibular reconstruction with the fibula free flap, Int J Oral Maxillofac Surg 46(8) (2017) 946-957. |  |
|  | [35] R. Sawh-Martinez, Y. Parsaei, R. Wu, A. Lin, P. Metzler, C. DeSesa, D.M. Steinbacher, Improved Temporomandibular Joint Position After 3-Dimensional Planned Mandibular Reconstruction, J Oral Maxillofac Surg 75(1) (2017) 197-206. |  |
|  | [36] G. Dell'Aversana Orabona, V. Abbate, F. Maglitto, P. Bonavolontà, G. Salzano, A. Romano, A. Reccia, U. Committeri, G. Iaconetta, L. Califano, Low-cost, self-made CAD/CAM-guiding system for mandibular reconstruction, Surg Oncol 27(2) (2018) 200-207. |  |
|  | [37] A. Dupret-Bories, S. Vergez, T. Meresse, F. Brouillet, G. Bertrand, Contribution of 3D printing to mandibular reconstruction after cancer, Eur Ann Otorhinolaryngol Head Neck Dis 135(2) (2018) 133-136. |  |
|  | [38] B. Jacek, P. Maciej, P. Tomasz, B. Agata, K. Wiesław, W. Radosław, G. Filip, 3D printed models in mandibular reconstruction with bony free flaps, J Mater Sci Mater Med 29(3) (2018) 23. |  |
|  | [39] J.I. Kass, E. Prisman, B.A. Miles, Guide design in virtual planning for scapular tip free flap reconstruction, Laryngoscope Investig Otolaryngol 3(3) (2018) 162-168. |  |
|  | [40] Z.H. Lee, T. Avraham, C. Monaco, A.A. Patel, D.L. Hirsch, J.P. Levine, Optimizing Functional Outcomes in Mandibular Condyle Reconstruction With the Free Fibula Flap Using Computer-Aided Design and Manufacturing Technology, J Oral Maxillofac Surg 76(5) (2018) 1098-1106. |  |
|  | [41] D. Pauchet, J.L. Pigot, F. Chabolle, C.A. Bach, Prefabricated fibula free flap with dental implants for mandibular reconstruction, Eur Ann Otorhinolaryngol Head Neck Dis 135(4) (2018) 279-282. |  |
|  | [42] W. Ren, L. Gao, S. Li, C. Chen, F. Li, Q. Wang, Y. Zhi, J. Song, Z. Dou, L. Xue, K. Zhi, Virtual Planning and 3D printing modeling for mandibular reconstruction with fibula free flap, Med Oral Patol Oral Cir Bucal 23(3) (2018) e359-e366. |  |
|  | [43] F.A.E. Smithers, K. Cheng, R. Jayaram, P. Mukherjee, J.R. Clark, Maxillofacial reconstruction using in-house virtual surgical planning, ANZ J Surg 88(9) (2018) 907-912. |  |
|  | [44] J. Blanc, C. Fuchsmann, V. Nistiriuc-Muntean, P. Jacquenot, P. Philouze, P. Ceruse, Evaluation of virtual surgical planning systems and customized devices in fibula free flap mandibular reconstruction, Eur Arch Otorhinolaryngol 276(12) (2019) 3477-3486. |  |
|  | [45] C. Spaas, O. Lenssen, Economic analysis of a low-cost virtual surgical planning protocol for mandibular reconstruction: a case series, Br J Oral Maxillofac Surg 57(8) (2019) 743-748. |  |
|  | [46] M. Zhang, P. Rao, D. Xia, L. Sun, X. Cai, J. Xiao, Functional Reconstruction of Mandibular Segment Defects With Individual Preformed Reconstruction Plate and Computed Tomographic Angiography-Aided Iliac Crest Flap, J Oral Maxillofac Surg 77(6) (2019) 1293-1304. |  |
|  | [47] L. Zheng, W. Wu, Y. Shi, J. Zhang, Mandibular Reconstruction With a Deep Circumflex Iliac Artery Flap Using Computer-Assisted and Intraoral Anastomosis Techniques, J Oral Maxillofac Surg 77(12) (2019) 2567-2572. |  |
|  | [48] M. Zho, Z. Shao, Y. Zhu, B. Liu, T. Wu, Comparison of Complicated and Simple Guiding Templates in Mandibular Reconstruction Using Vascularized Iliac Crest Flap, Biomed Res Int 2019 (2019) 7496538. |  |
|  | [49] H. Abo Sharkh, N. Makhoul, In-House Surgeon-Led Virtual Surgical Planning for Maxillofacial Reconstruction, J Oral Maxillofac Surg 78(4) (2020) 651-660. |  |
|  | [50] A. Damecourt, N. Nieto, S. Galmiche, R. Garrel, M. de Boutray, In-house 3D treatment planning for mandibular reconstruction by free fibula flap in cancer: Our technique, Eur Ann Otorhinolaryngol Head Neck Dis 137(6) (2020) 501-505. |  |
|  | [51] M. Lv, X. Yang, A. Gupta, Y. Shen, J. Li, J. Sun, Sequential application of novel guiding plate system for accurate transoral mandibular reconstruction, Oral Oncol 111 (2020) 104846. |  |
|  | [52] S. Mahendru, R. Jain, A. Aggarwal, H.S. Aulakh, A. Jain, R.K. Khazanchi, D. Sarin, CAD-CAM vs conventional technique for mandibular reconstruction with free fibula flap: A comparison of outcomes, Surg Oncol 34 (2020) 284-291. |  |
|  | [53] T. Numajiri, D. Morita, R. Yamochi, H. Nakamura, S. Tsujiko, Y. Sowa, K. Toyoda, T. Tsujikawa, A. Arai, S. Hirano, Does an In-House Computer-Aided Design/Computer-Aided Manufacturing Approach Contribute to Accuracy and Time Shortening in Mandibular Reconstruction?, J Craniofac Surg 31(7) (2020) 1928-1932. |  |
|  | [54] S. Shen, C. Yang, J. Wu, L. Zhang, X. Wang, J. Shi, S. Zhang, A Modified Method Using Double Computed Tomography Scan Procedure to Maintain Mandibular Width in Mandibular Reconstruction, J Craniofac Surg 31(2) (2020) e126-e130. |  |
|  | [55] E. Wang, J.S. Durham, D.W. Anderson, E. Prisman, Clinical evaluation of an automated virtual surgical planning platform for mandibular reconstruction, Head Neck 42(12) (2020) 3506-3514. |  |
|  | [56] E. Zavattero, M. Fasolis, A. Novaresio, G. Gerbino, C. Borbon, G. Ramieri, The Shape of Things to Come: In-Hospital Three-Dimensional Printing for Mandibular Reconstruction Using Fibula Free Flap, Laryngoscope 130(12) (2020) E811-e816. |  |
|  | [57] S. Bartier, O. Mazzaschi, L. Benichou, E. Sauvaget, Computer-assisted versus traditional technique in fibular free-flap mandibular reconstruction: A CT symmetry study, Eur Ann Otorhinolaryngol Head Neck Dis 138(1) (2021) 23-27. |  |
|  | [58] J. Moe, J. Foss, R. Herster, C. Powell, J. Helman, B.B. Ward, K. VanKoevering, An In-House Computer-Aided Design and Computer-Aided Manufacturing Workflow for Maxillofacial Free Flap Reconstruction is Associated With a Low Cost and High Accuracy, J Oral Maxillofac Surg 79(1) (2021) 227-236. |  |
|  | [59] J.E. O'Connell, A.G. Schache, S. Fleming, R.J. Shaw, Virtual surgical planning in mandibular reconstruction using scapular free flaps: a technical note, Br J Oral Maxillofac Surg 59(6) (2021) 724-725. |  |
|  | [60] A. Ohkoshi, N. Sato, K. Kurosawa, H. Miyashita, R. Ishii, A. Nakanome, T. Ogawa, M. Tachi, T. Takahashi, Y. Katori, Impact of CAD/CAM mandibular reconstruction on chewing and swallowing function after surgery for locally advanced oral cancer: A retrospective study of 50 cases, Auris Nasus Larynx 48(5) (2021) 1007-1012. |  |
|  | [61] L. Šimić, V. Kopačin, I. Mumlek, J. Butković, V. Zubčić, Improved technique of personalised surgical guides generation for mandibular free flap reconstruction using an open-source tool, Eur Radiol Exp 5(1) (2021) 30. |  |
|  | [62] P. Wu, L. Hu, H. Li, L. Feng, Y. Liu, S. Zhang, X.C. Li, M.L. Zhang, S.Y. Yang, R.J. Lu, Clinical application and accuracy analysis of 3D printing guide plate based on polylactic acid in mandible reconstruction with fibula flap, Ann Transl Med 9(6) (2021) 460. |  |
|  | [63] Z. Zhu, Z. He, Y. Tai, Y. Liu, H. Liu, E. Luo, Surgical Guides and Prebent Titanium Improve the Planning for the Treatment of Dentofacial Deformities Secondary to Condylar Osteochondroma, J Craniofac Surg (2021). |  |
|  | [64] M. Cedillo, S. Córdova, S. Larralde, F. Martínez, F. Sandoval, F. Suntaxi, A Comparative Study of Analog Preoperative Planning Versus Virtual Preoperative Planning for Mandibular Reconstruction With Fibula Free Flap, J Craniofac Surg (2022). |  |
|  | [65] C.H. Lin, C.H. Hsu, K. Adarsh, C.M. Hsu, C.M. Wu, Real-time intraoperative computed tomography can accurize virtual surgical planning on the double-barrel fibular flap for mandibular reconstruction, J Plast Reconstr Aesthet Surg 75(8) (2022) 2702-2705. |  |
| **Cutting guides + prebent surgical plates (registered positions)** | | **6** |
|  | [1] F. Wilde, K. Winter, K. Kletsch, K. Lorenz, A. Schramm, Mandible reconstruction using patient-specific pre-bent reconstruction plates: comparison of standard and transfer key methods, Int J Comput Assist Radiol Surg 10(2) (2015) 129-40. |  |
|  | [2] Y. Liang, C. Jiang, L. Wu, W. Wang, Y. Liu, X. Jian, Application of Combined Osteotomy and Reconstruction Pre-Bent Plate Position (CORPPP) Technology to Assist in the Precise Reconstruction of Segmental Mandibular Defects, J Oral Maxillofac Surg 75(9) (2017) 2026.e1-2026.e10. |  |
|  | [3] J. Chen, R. Zhang, Y. Liang, Y. Ma, S. Song, C. Jiang, Deviation Analyses of Computer-Assisted, Template-Guided Mandibular Reconstruction With Combined Osteotomy and Reconstruction Pre-Shaped Plate Position Technology: A Comparative Study, Front Oncol 11 (2021) 719466. |  |
|  | [4] S. Qiu, Y. Kang, M. Ding, H. Zhu, Y. Zhang, L. Zhang, X. Shan, Z. Cai, Mandibular Reconstruction With the Iliac Flap Under the Guidance of A Series of Digital Surgical Guides, J Craniofac Surg 32(5) (2021) 1777-1779. |  |
|  | [5] B. Khatib, M. Couey, A. Patel, A. Cheng, R.B. Bell, "Custom" Plate in a Day-Accurate Predictive Hole Fabrication Using Point-of-Care 3-Dimensional Printing, J Oral Maxillofac Surg 80(3) (2022) 559-568. |  |
|  | [6] Y. Lai, C. Wang, C. Mao, M. Lu, Q. Ouyang, Y. Fang, Z. Cai, W. Chen, Mandible reconstruction with free fibula flaps: Accuracy of a cost-effective modified semicomputer-assisted surgery compared with computer-assisted surgery - A retrospective study, J Craniomaxillofac Surg 50(3) (2022) 274-280. |  |
| **Cutting guides + positioning guides + prebent surgical plates** | | **17** |
|  | [1] A. Abou-ElFetouh, A. Barakat, K. Abdel-Ghany, Computer-guided rapid-prototyped templates for segmental mandibular osteotomies: a preliminary report, Int J Med Robot 7(2) (2011) 187-92. |  |
|  | [2] G.S. Zheng, Y.X. Su, G.Q. Liao, Z.F. Chen, L. Wang, P.F. Jiao, H.C. Liu, Y.Q. Zhong, T.H. Zhang, Y.J. Liang, Mandible reconstruction assisted by preoperative virtual surgical simulation, Oral Surg Oral Med Oral Pathol Oral Radiol 113(5) (2012) 604-11. |  |
|  | [3] G.S. Zheng, Y.X. Su, G.Q. Liao, P.F. Jiao, L.Z. Liang, S.E. Zhang, H.C. Liu, Mandible reconstruction assisted by preoperative simulation and transferring templates: cadaveric study of accuracy, J Oral Maxillofac Surg 70(6) (2012) 1480-5. |  |
|  | [4] G.S. Zheng, Y.X. Su, G.Q. Liao, H.C. Liu, S.E. Zhang, L.Z. Liang, Mandibular reconstruction assisted by preoperative simulation and accurate transferring templates: preliminary report of clinical application, J Oral Maxillofac Surg 71(9) (2013) 1613-8. |  |
|  | [5] Y.F. Liu, L.W. Xu, H.Y. Zhu, S.S. Liu, Technical procedures for template-guided surgery for mandibular reconstruction based on digital design and manufacturing, Biomed Eng Online 13 (2014) 63. |  |
|  | [6] R. Chen, H.Q. Zhang, Z.X. Huang, S.H. Li, D.M. Zhang, Z.Q. Huang, Computer-assisted resection and reconstruction of bilateral osteoradionecrosis of the mandible using 2 separate flaps prepared from a single fibula, Oral Surg Oral Med Oral Pathol Oral Radiol 126(2) (2018) 102-106. |  |
|  | [7] J. Geusens, Y. Sun, H.T. Luebbers, M. Bila, V. Darche, C. Politis, Accuracy of Computer-Aided Design/Computer-Aided Manufacturing-Assisted Mandibular Reconstruction With a Fibula Free Flap, J Craniofac Surg 30(8) (2019) 2319-2323. |  |
|  | [8] F. Goormans, Y. Sun, M. Bila, J. Schoenaers, J. Geusens, H.T. Lübbers, W. Coucke, C. Politis, Accuracy of computer-assisted mandibular reconstructions with free fibula flap: Results of a single-center series, Oral Oncol 97 (2019) 69-75. |  |
|  | [9] I.M. Elsharabasy, H. Elhafez, S.A.E. Ahmed, W.M. Ayad, Evaluation of the Accuracy of Three-Dimensional Virtual Surgical Planning for Reconstruction of Mandibular Defects Using Free Fibular Flap, J Craniofac Surg 31(4) (2020) 950-955. |  |
|  | [10] L. Han, X. Zhang, Z. Guo, J. Long, Application of optimized digital surgical guides in mandibular resection and reconstruction with vascularized fibula flaps: Two case reports, Medicine (Baltimore) 99(35) (2020) e21942. |  |
|  | [11] Y. Li, Z. Shao, Y. Zhu, B. Liu, T. Wu, Virtual Surgical Planning for Successful Second-Stage Mandibular Defect Reconstruction Using Vascularized Iliac Crest Bone Flap: A Valid and Reliable Method, Ann Plast Surg 84(2) (2020) 183-187. |  |
|  | [12] T. Lu, Z. Shao, B. Liu, T. Wu, Recent advance in patient-specific 3D printing templates in mandibular reconstruction, J Mech Behav Biomed Mater 106 (2020) 103725. |  |
|  | [13] H. Ma, S. Shujaat, M. Bila, Y. Sun, J. Vranckx, C. Politis, R. Jacobs, Computer-assisted versus traditional freehand technique for mandibular reconstruction with free vascularized fibular flap: A matched-pair study, J Plast Reconstr Aesthet Surg 74(11) (2021) 3031-3039. |  |
|  | [14] H. Ma, S. Shujaat, J. Van Dessel, Y. Sun, M. Bila, J. Vranckx, C. Politis, R. Jacobs, Adherence to Computer-Assisted Surgical Planning in 136 Maxillofacial Reconstructions, Front Oncol 11 (2021) 713606. |  |
|  | [15] M.M. May, B.M. Howe, T.J. O'Byrne, A.E. Alexander, J.M. Morris, E.J. Moore, J.L. Kasperbauer, J.R. Janus, K.M. Van Abel, H.J. Dickens, D.L. Price, Short and long-term outcomes of three-dimensional printed surgical guides and virtual surgical planning versus conventional methods for fibula free flap reconstruction of the mandible: Decreased nonunion and complication rates, Head Neck 43(8) (2021) 2342-2352. |  |
|  | [16] L.M. Ritschl, P. Kilbertus, F.D. Grill, M. Schwarz, J. Weitz, M. Nieberler, K.D. Wolff, A.M. Fichter, In-House, Open-Source 3D-Software-Based, CAD/CAM-Planned Mandibular Reconstructions in 20 Consecutive Free Fibula Flap Cases: An Explorative Cross-Sectional Study With Three-Dimensional Performance Analysis, Front Oncol 11 (2021) 731336. |  |
|  | [17] C. Wang, Z. Meng, B. Jie, R. Li, Y. He, M. Xu, Accurate Reconstruction of Mandibular Defects With Vascularized Bone Flaps Through Utilization of Mandible Space-Retention Guides, J Craniofac Surg (2022). |  |
| **Cutting guides + PSSPs** | | **65** |
|  | [1] L. Ciocca, S. Mazzoni, M. Fantini, C. Marchetti, R. Scotti, The design and rapid prototyping of surgical guides and bone plates to support iliac free flaps for mandible reconstruction, Plast Reconstr Surg 129(5) (2012) 859e-861e. |  |
|  | [2] L. Ciocca, S. Mazzoni, M. Fantini, F. Persiani, P. Baldissara, C. Marchetti, R. Scotti, A CAD/CAM-prototyped anatomical condylar prosthesis connected to a custom-made bone plate to support a fibula free flap, Med Biol Eng Comput 50(7) (2012) 743-9. |  |
|  | [3] L. Ciocca, S. Mazzoni, M. Fantini, F. Persiani, C. Marchetti, R. Scotti, CAD/CAM guided secondary mandibular reconstruction of a discontinuity defect after ablative cancer surgery, J Craniomaxillofac Surg 40(8) (2012) e511-5. |  |
|  | [4] S. Mazzoni, C. Marchetti, R. Sgarzani, R. Cipriani, R. Scotti, L. Ciocca, Prosthetically guided maxillofacial surgery: evaluation of the accuracy of a surgical guide and custom-made bone plate in oncology patients after mandibular reconstruction, Plast Reconstr Surg 131(6) (2013) 1376-1385. |  |
|  | [5] A. Tarsitano, S. Mazzoni, R. Cipriani, R. Scotti, C. Marchetti, L. Ciocca, The CAD-CAM technique for mandibular reconstruction: an 18 patients oncological case-series, J Craniomaxillofac Surg 42(7) (2014) 1460-4. |  |
|  | [6] F. Wilde, C.P. Cornelius, A. Schramm, Computer-Assisted Mandibular Reconstruction using a Patient-Specific Reconstruction Plate Fabricated with Computer-Aided Design and Manufacturing Techniques, Craniomaxillofac Trauma Reconstr 7(2) (2014) 158-66. |  |
|  | [7] L. Ciocca, C. Marchetti, S. Mazzoni, P. Baldissara, M.R. Gatto, R. Cipriani, R. Scotti, A. Tarsitano, Accuracy of fibular sectioning and insertion into a rapid-prototyped bone plate, for mandibular reconstruction using CAD-CAM technology, J Craniomaxillofac Surg 43(1) (2015) 28-33. |  |
|  | [8] C.P. Cornelius, W. Smolka, G.A. Giessler, F. Wilde, F.A. Probst, Patient-specific reconstruction plates are the missing link in computer-assisted mandibular reconstruction: A showcase for technical description, J Craniomaxillofac Surg 43(5) (2015) 624-9. |  |
|  | [9] R.H. Schepers, G.M. Raghoebar, A. Vissink, M.W. Stenekes, J. Kraeima, J.L. Roodenburg, H. Reintsema, M.J. Witjes, Accuracy of fibula reconstruction using patient-specific CAD/CAM reconstruction plates and dental implants: A new modality for functional reconstruction of mandibular defects, J Craniomaxillofac Surg 43(5) (2015) 649-57. |  |
|  | [10] A. Tarsitano, L. Ciocca, R. Cipriani, R. Scotti, C. Marchetti, Mandibular reconstruction using fibula free flap harvested using a customised cutting guide: how we do it, Acta Otorhinolaryngol Ital 35(3) (2015) 198-201. |  |
|  | [11] A. Tarsitano, G. Del Corso, L. Ciocca, R. Scotti, C. Marchetti, Mandibular reconstructions using computer-aided design/computer-aided manufacturing: A systematic review of a defect-based reconstructive algorithm, J Craniomaxillofac Surg 43(9) (2015) 1785-91. |  |
|  | [12] F. Wilde, H. Hanken, F. Probst, A. Schramm, M. Heiland, C.P. Cornelius, Multicenter study on the use of patient-specific CAD/CAM reconstruction plates for mandibular reconstruction, Int J Comput Assist Radiol Surg 10(12) (2015) 2035-51. |  |
|  | [13] M. Berrone, E. Crosetti, P.L. Tos, M. Pentenero, G. Succo, Fibular osteofasciocutaneous flap in computer-assisted mandibular reconstruction: technical aspects in oral malignancies, Acta Otorhinolaryngol Ital 36(6) (2016) 469-478. |  |
|  | [14] L. Ciocca, A. Tarsitano, C. Marchetti, R. Scotti, A CAD-CAM-prototyped temporomandibular condyle connected to a bony plate to support a free fibula flap in patients undergoing mandiblectomy: A pilot study with 5 years of follow up, J Craniomaxillofac Surg 44(7) (2016) 811-9. |  |
|  | [15] C.P. Cornelius, G.A. Giessler, F. Wilde, M.C. Metzger, G. Mast, F.A. Probst, Iterations of computer- and template assisted mandibular or maxillary reconstruction with free flaps containing the lateral scapular border--Evolution of a biplanar plug-on cutting guide, J Craniomaxillofac Surg 44(3) (2016) 229-41. |  |
|  | [16] C. Monaco, J.T. Stranix, T. Avraham, L. Brecht, P.B. Saadeh, D. Hirsch, J.P. Levine, Evolution of surgical techniques for mandibular reconstruction using free fibula flaps: The next generation, Head Neck 38 Suppl 1 (2016) E2066-73. |  |
|  | [17] M. Qaisi, H. Kolodney, G. Swedenburg, R. Chandran, R. Caloss, Fibula Jaw in a Day: State of the Art in Maxillofacial Reconstruction, J Oral Maxillofac Surg 74(6) (2016) 1284.e1-1284.e15. |  |
|  | [18] S. Battaglia, V. Maiolo, G. Savastio, M. Zompatori, F. Contedini, E. Antoniazzi, R. Cipriani, C. Marchetti, A. Tarsitano, Osteomyocutaneous fibular flap harvesting: Computer-assisted planning of perforator vessels using Computed Tomographic Angiography scan and cutting guide, J Craniomaxillofac Surg 45(10) (2017) 1681-1686. |  |
|  | [19] T. De Maesschalck, D.S. Courvoisier, P. Scolozzi, Computer-assisted versus traditional freehand technique in fibular free flap mandibular reconstruction: a morphological comparative study, Eur Arch Otorhinolaryngol 274(1) (2017) 517-526. |  |
|  | [20] F. Mascha, K. Winter, S. Pietzka, M. Heufelder, A. Schramm, F. Wilde, Accuracy of computer-assisted mandibular reconstructions using patient-specific implants in combination with CAD/CAM fabricated transfer keys, J Craniomaxillofac Surg 45(11) (2017) 1884-1897. |  |
|  | [21] A. Tarsitano, S. Battaglia, V. Ramieri, P. Cascone, L. Ciocca, R. Scotti, C. Marchetti, Short-term outcomes of mandibular reconstruction in oncological patients using a CAD/CAM prosthesis including a condyle supporting a fibular free flap, J Craniomaxillofac Surg 45(2) (2017) 330-337. |  |
|  | [22] A.M. Hoving, J. Kraeima, R.H. Schepers, H. Dijkstra, J.H. Potze, B. Dorgelo, M.J.H. Witjes, Optimisation of three-dimensional lower jaw resection margin planning using a novel Black Bone magnetic resonance imaging protocol, PLoS One 13(4) (2018) e0196059. |  |
|  | [23] F. Iglesias-Martín, L.G. Oliveros-López, A. Fernández-Olavarría, M.A. Serrera-Figallo, A. Gutiérrez-Corrales, D. Torres-Lagares, J.L. Gutiérrez-Pérez, Advantages of surgical simulation in the surgical reconstruction of oncological patients, Med Oral Patol Oral Cir Bucal 23(5) (2018) e596-e601. |  |
|  | [24] J. Kraeima, B. Dorgelo, H.A. Gulbitti, R. Steenbakkers, K.P. Schepman, J.L.N. Roodenburg, F.K.L. Spijkervet, R.H. Schepers, M.J.H. Witjes, Multi-modality 3D mandibular resection planning in head and neck cancer using CT and MRI data fusion: A clinical series, Oral Oncol 81 (2018) 22-28. |  |
|  | [25] A. Tarsitano, S. Battaglia, F. Ricotta, B. Bortolani, L. Cercenelli, E. Marcelli, R. Cipriani, C. Marchetti, Accuracy of CAD/CAM mandibular reconstruction: A three-dimensional, fully virtual outcome evaluation method, J Craniomaxillofac Surg 46(7) (2018) 1121-1125. |  |
|  | [26] W.F. Yang, W.S. Choi, Y.Y. Leung, J.P. Curtin, R. Du, C.Y. Zhang, X.S. Chen, Y.X. Su, Three-dimensional printing of patient-specific surgical plates in head and neck reconstruction: A prospective pilot study, Oral Oncol 78 (2018) 31-36. |  |
|  | [27] D. Zweifel, M.G. Bredell, H. Essig, T. Gander, M. Lanzer, C. Rostetter, M. Rücker, S. Studer, Total virtual workflow in CAD-CAM bony reconstruction with a single step free fibular graft and immediate dental implants, Br J Oral Maxillofac Surg 56(9) (2018) 859-863. |  |
|  | [28] S. Battaglia, F. Ricotta, V. Maiolo, G. Savastio, F. Contedini, R. Cipriani, B. Bortolani, L. Cercenelli, E. Marcelli, C. Marchetti, A. Tarsitano, Computer-assisted surgery for reconstruction of complex mandibular defects using osteomyocutaneous microvascular fibular free flaps: Use of a skin paddle-outlining guide for soft-tissue reconstruction. A technical report, J Craniomaxillofac Surg 47(2) (2019) 293-299. |  |
|  | [29] M. Davey, N.M. McInerney, T. Barry, A. Hussey, S. Potter, Virtual Surgical Planning Computer-aided Design-guided Osteocutaneous Fibular Free Flap for Craniofacial Reconstruction: A Novel Surgical Approach, Cureus 11(11) (2019) e6256. |  |
|  | [30] H.S. Ong, J.N. Liu, A. Ahmed, X.Z. Qu, K. Wan, D.P. Xie, C.P. Zhang, Improved accuracy of hemimandibular reconstructions involving the condyle by utilizing hydroformed reconstruction plates rather than hand-bent stock plates, Head Neck 41(9) (2019) 3168-3176. |  |
|  | [31] A. Patel, P. Harrison, A. Cheng, B. Bray, R.B. Bell, Fibular Reconstruction of the Maxilla and Mandible with Immediate Implant-Supported Prosthetic Rehabilitation: Jaw in a Day, Oral Maxillofac Surg Clin North Am 31(3) (2019) 369-386. |  |
|  | [32] M.H. Smith, C.H. Schrag, S.P. Chandarana, J.G. Cobb, T.W. Matthews, C.D. McKenzie, J.L. Matthews, Novel Plate Design to Improve Mandibular and Maxillary Reconstruction with the Osteocutaneous Fibula Flap, Plast Reconstr Surg Glob Open 7(1) (2019) e2094. |  |
|  | [33] D. Zweifel, M.G. Bredell, M. Lanzer, C. Rostetter, M. Rücker, S. Studer, Precision of Simultaneous Guided Dental Implantation in Microvascular Fibular Flap Reconstructions With and Without Additional Guiding Splints, J Oral Maxillofac Surg 77(5) (2019) 971-976. |  |
|  | [34] M. Berrone, E. Crosetti, B. Battiston, G. Succo, Virtual Surgical Planning for Mandible Reconstruction With a Double Barrel Fibula Flap and Immediate Implant Placement, J Craniofac Surg 31(1) (2020) e41-e43. |  |
|  | [35] S. Dahake, A. Kuthe, M. Mawale, P. Sapkal, A. Bagde, S. Daronde, M. Kamble, B. Sarode, Development of customized implant and customized surgical osteotomy guide in ablative tumor surgery for accurate mandibular reconstruction, Int J Med Robot 16(1) (2020) e2046. |  |
|  | [36] Ł. Krakowczyk, A. Piotrowska-Seweryn, C. Szymczyk, J. Wierzgoń, K. Oleś, R. Ulczok, K. Donocik, K. Dowgierd, A. Maciejewski, Virtual surgical planning and cone beam computed tomography in reconstruction of head and neck tumors - pilot study, Otolaryngol Pol 75(2) (2020) 28-33. |  |
|  | [37] Z.H. Lee, A.R. Alfonso, E.P. Ramly, R.S. Kantar, J.W. Yu, D. Daar, D.L. Hirsch, A. Jacobson, J.P. Levine, The Latest Evolution in Virtual Surgical Planning: Customized Reconstruction Plates in Free Fibula Flap Mandibular Reconstruction, Plast Reconstr Surg 146(4) (2020) 872-879. |  |
|  | [38] H. Patel, N. Saadat, A.S. Ho, J. Mallen-St Clair, Virtual Surgical Planning for Bisphosphonate-Related Osteonecrosis of the Jaw: A Valuable Application in Advanced Cases, Cureus 12(8) (2020) e9696. |  |
|  | [39] T. Seier, L. Hingsammer, P. Schumann, T. Gander, M. Rücker, M. Lanzer, Virtual planning, simultaneous dental implantation and CAD/CAM plate fixation: a paradigm change in maxillofacial reconstruction, Int J Oral Maxillofac Surg 49(7) (2020) 854-861. |  |
|  | [40] D.C. Sukato, D. Hammer, W. Wang, T. Shokri, F. Williams, Y. Ducic, Experience With "Jaw in a Day" Technique, J Craniofac Surg 31(5) (2020) 1212-1217. |  |
|  | [41] A.M. Weyh, A. Quimby, R.P. Fernandes, Three-Dimensional Computer-Assisted Surgical Planning and Manufacturing in Complex Mandibular Reconstruction, Atlas Oral Maxillofac Surg Clin North Am 28(2) (2020) 145-150. |  |
|  | [42] W.F. Yang, W.S. Choi, W.Y. Zhu, Y.X. Su, "One-piece" patient-specific reconstruction plate for double-barrel fibula-based mandibular reconstruction, Int J Oral Maxillofac Surg 49(8) (2020) 1016-1019. |  |
|  | [43] W.F. Yang, C.Y. Zhang, W.S. Choi, W.Y. Zhu, D.T.S. Li, X.S. Chen, R. Du, Y.X. Su, A novel 'surgeon-dominated' approach to the design of 3D-printed patient-specific surgical plates in mandibular reconstruction: a proof-of-concept study, Int J Oral Maxillofac Surg 49(1) (2020) 13-21. |  |
|  | [44] H.Q. Zhang, Q.X. Li, Y.Y. Wang, Z.S. Wang, Z.Y. Lin, L.J. Sha, D.M. Zhang, J. Liu, J.J. Wang, J.S. Li, S. Fan, Combination of biomechanical evaluation and accurate placement of dental implants: a new concept of virtual surgery in maxillary and mandibular functional reconstruction, Br J Oral Maxillofac Surg 58(1) (2020) 62-68. |  |
|  | [45] R. Antúnez-Conde, J.I. Salmerón, A. Díez-Montiel, M. Agea, D. Gascón, Á. Sada, I. Navarro Cuéllar, M. Tousidonis, S. Ochandiano, G. Arenas, C. Navarro Cuéllar, Mandibular Reconstruction With Fibula Flap and Dental Implants Through Virtual Surgical Planning and Three Different Techniques: Double-Barrel Flap, Implant Dynamic Navigation and CAD/CAM Mesh With Iliac Crest Graft, Front Oncol 11 (2021) 719712. |  |
|  | [46] J. Chauvel-Picard, K. Kreutzer, M. Heiland, T. Kreusch, T. Ebker, B. Beck-Broichsitter, One stage microvascular mandible reconstruction by using scapula chimeric flap combined with computer-aided-design and computer-aided-manufacturing plate including bilateral alloplastic TMJ prosthesis: A case report, Microsurgery 41(3) (2021) 263-269. |  |
|  | [47] E. Crosetti, G. Succo, B. Battiston, F. D'Addabbo, M. Tascone, E. Maldi, I. Bertotto, M. Berrone, Surgical Margins After Computer-Assisted Mandibular Reconstruction: A Retrospective Study, Front Oral Health 2 (2021) 806477. |  |
|  | [48] G.W. Jenkins, S. Iqbal, N. West, I. Ellabban, M.P. Kennedy, J.R. Adams, Dosimetry-guided virtual surgical planning in the reconstruction of mandibular osteoradionecrosis, Br J Oral Maxillofac Surg 59(8) (2021) 947-951. |  |
|  | [49] D. Leinkram, J. Wykes, C. Palme, S. Deshpande, M. McLaughlin, P. Garg, C. Wallace, D. Howes, J.R. Clark, Occlusal-based planning for dental rehabilitation following segmental resection of the mandible and maxilla, ANZ J Surg 91(3) (2021) 451-452. |  |
|  | [50] G.L. Lilly, D. Petrisor, M.K. Wax, Mandibular rehabilitation: From the Andy Gump deformity to jaw-in-a-day, Laryngoscope Investig Otolaryngol 6(4) (2021) 708-720. |  |
|  | [51] S. Maniskas, N. Pourtaheri, L. Chandler, X. Lu, K.C. Bruckman, D.M. Steinbacher, Conformity of the Virtual Surgical Plan to the Actual Result Comparing Five Craniofacial Procedure Types, Plast Reconstr Surg 147(4) (2021) 915-924. |  |
|  | [52] P.L. Myers, J.A. Nelson, E.B. Rosen, R.J. Allen, Jr., J.J. Disa, E. Matros, Virtual Surgical Planning for Oncologic Mandibular and Maxillary Reconstruction, Plast Reconstr Surg Glob Open 9(9) (2021) e3672. |  |
|  | [53] W.F. Yang, W.S. Choi, M.C. Wong, W. Powcharoen, W.Y. Zhu, J.K. Tsoi, M. Chow, K.W. Kwok, Y.X. Su, Three-Dimensionally Printed Patient-Specific Surgical Plates Increase Accuracy of Oncologic Head and Neck Reconstruction Versus Conventional Surgical Plates: A Comparative Study, Ann Surg Oncol 28(1) (2021) 363-375. |  |
|  | [54] W.F. Yang, W.S. Choi, W.Y. Zhu, C.Y. Zhang, D.T.S. Li, J.K. Tsoi, A.W. Tang, K.W. Kwok, Y.X. Su, Spatial deviations of the temporomandibular joint after oncological mandibular reconstruction, Int J Oral Maxillofac Surg (2021). |  |
|  | [55] E. Zavattero, A. Bolzoni, G. Dell'Aversana, M. Santagata, O. Massarelli, A. Ferri, M. Della Monaca, C. Copelli, M. Gessaroli, S. Valsecchi, C. Borbon, G.A. Beltramini, G. Ramieri, V. Valentini, G.P. Tartaro, R. Cocchi, A. Varazzani, L. Califano, A. Baj, Accuracy of Fibula Reconstruction Using Patient-Specific Cad/Cam Plates: A Multicenter Study on 47 Patients, Laryngoscope 131(7) (2021) E2169-e2175. |  |
|  | [56] W.Y. Zhu, W.S. Choi, Y.X. Su, Three-dimensional Printing Technology for Deep Circumflex Iliac Artery Flap: From Recipient to Donor Sites, Plast Reconstr Surg Glob Open 9(6) (2021) e3618. |  |
|  | [57] D.J. Byun, D.A. Daar, K. Spuhler, L. Anzai, L. Witek, D. Barbee, A.S. Jacobson, J.P. Levine, K.S. Hu, Osteoradionecrosis After Radiation to Reconstructed Mandible With Titanium Plate and Osseointegrated Dental Implants, Pract Radiat Oncol 12(2) (2022) 90-94. |  |
|  | [58] K. Dowgierd, R. Pokrowiecki, W. Wolanski, E. Kawlewska, M. Kozakiewicz, J. Wos, M. Dowgierd, Ł. Krakowczyk, Analysis of the effects of mandibular reconstruction based on microvascular free flaps after oncological resections in 21 patients, using 3D planning, surgical templates and individual implants, Oral Oncol 127 (2022) 105800. |  |
|  | [59] P. Gennaro, F. Cascino, L.V. Pignataro, G. Gabriele, Deviation analysis in custom-made mandibular reconstruction: how to evaluate results, BMJ Case Rep 15(7) (2022). |  |
|  | [60] M.J.L. Hurrell, J. Singh, D. Leinkram, J.R. Clark, Patient specific implant with high condylar neck osteotomy for temporomandibular joint preservation in segmental mandibulectomy, Oral Oncol 134 (2022) 106084. |  |
|  | [61] K. Itamura, S. Kupferman, J. Lee, J. Mallen-St Clair, Jaw-in-a-Riley-Day: Mandibular Free Flap Reconstruction With Virtual Surgical Planning in a Patient With Familial Dysautonomia, Cureus 14(6) (2022) e26336. |  |
|  | [62] A. Mc Goodson, C. Thomas, L. Maxwell, P.A. Brennan, E.M. Williams, The 3D-printed miniplate-jig system: a new, rapid, precise, and user-friendly approach to miniplate fixation of free-tissue mandibular reconstructions, Br J Oral Maxillofac Surg (2022). |  |
|  | [63] D.B. Powers, J. Breeze, D. Erdmann, Vascularized Fibula TMJ Reconstruction: A Report of Five Cases featuring Computerized Patient-specific Surgical Planning, Plast Reconstr Surg Glob Open 10(8) (2022) e4465. |  |
|  | [64] J. Shum, V. Manon, A. Huang, A Combined Transoral and Contralateral Submandibular Approach in the Surgical Management of Osteoradionecrosis of the Mandible With Free Flap Reconstruction, J Oral Maxillofac Surg (2022). |  |
|  | [65] W.F. Yang, W.S. Choi, W.Y. Zhu, C.Y. Zhang, D.T.S. Li, J.K. Tsoi, A.W. Tang, K.W. Kwok, Y.X. Su, Spatial deviations of the temporomandibular joint after oncological mandibular reconstruction, Int J Oral Maxillofac Surg 51(1) (2022) 44-53. |  |
| **Cutting guides + positioning guides + PSSPs** | | **1** |
|  | [1] H. Ma, J. Van Dessel, M. Bila, Y. Sun, C. Politis, R. Jacobs, Application of Three-Dimensional Printed Customized Surgical Plates for Mandibular Reconstruction: Report of Consecutive Cases and Long-Term Postoperative Evaluation, J Craniofac Surg 32(7) (2021) e663-e667. |  |
| **Navigation (only)** | | **10** |
|  | [1] P. Juergens, C. Klug, Z. Krol, J. Beinemann, H. Kim, M. Reyes, G. Guevara-Rojas, H.F. Zeilhofer, R. Ewers, K. Schicho, Navigation-guided harvesting of autologous iliac crest graft for mandibular reconstruction, J Oral Maxillofac Surg 69(11) (2011) 2915-23. |  |
|  | [2] M. Rana, H. Essig, A.M. Eckardt, F. Tavassol, M. Ruecker, A. Schramm, N.C. Gellrich, Advances and innovations in computer-assisted head and neck oncologic surgery, J Craniofac Surg 23(1) (2012) 272-8. |  |
|  | [3] W. Zhang, B. Li, H. Gui, L. Zhang, X. Wang, G. Shen, Reconstruction of complex mandibular defect with computer-aided navigation and orthognathic surgery, J Craniofac Surg 24(3) (2013) e229-33. |  |
|  | [4] H.B. Yu, B. Li, L. Zhang, S.G. Shen, X.D. Wang, Computer-assisted surgical planning and intraoperative navigation in the treatment of condylar osteochondroma, Int J Oral Maxillofac Surg 44(1) (2015) 113-8. |  |
|  | [5] X.F. Shan, H.M. Chen, J. Liang, J.W. Huang, L. Zhang, Z.G. Cai, C. Guo, Surgical navigation-assisted mandibular reconstruction with fibula flaps, Int J Oral Maxillofac Surg 45(4) (2016) 448-53. |  |
|  | [6] J. Wu, J. Sun, S.G. Shen, B. Xu, J. Li, S. Zhang, Computer-assisted navigation: its role in intraoperatively accurate mandibular reconstruction, Oral Surg Oral Med Oral Pathol Oral Radiol 122(2) (2016) 134-42. |  |
|  | [7] Y. Yu, W.B. Zhang, X.J. Liu, C.B. Guo, G.Y. Yu, X. Peng, A New Procedure Assisted by Digital Techniques for Secondary Mandibular Reconstruction With Free Fibula Flap, J Craniofac Surg 27(8) (2016) 2009-2014. |  |
|  | [8] Y. Yu, W.B. Zhang, X.J. Liu, C.B. Guo, G.Y. Yu, X. Peng, Three-Dimensional Accuracy of Virtual Planning and Surgical Navigation for Mandibular Reconstruction With Free Fibula Flap, J Oral Maxillofac Surg 74(7) (2016) 1503.e1-1503.e10. |  |
|  | [9] S.Y. Shen, Y. Yu, W.B. Zhang, X.J. Liu, X. Peng, Angle-to-Angle Mandibular Defect Reconstruction With Fibula Flap by Using a Mandibular Fixation Device and Surgical Navigation, J Craniofac Surg 28(6) (2017) 1486-1491. |  |
|  | [10] D. Bradley, T. Willson, J.B. Chang, B. Gandolfi, T.R. Zhu, J.P. Bradley, J.C. Lee, Intraoperative Three-dimensional Virtual Reality and Computed Tomographic Guidance in Temporomandibular Joint Arthroplasty of Syndromic Craniofacial Dysostoses, Plast Reconstr Surg Glob Open 7(9) (2019) e2388. |  |
| **Navigation + customized graft tray** | | **1** |
|  | [1] X.F. Shan, H.M. Chen, J. Liang, J.W. Huang, Z.G. Cai, Surgical Reconstruction of Maxillary and Mandibular Defects Using a Printed Titanium Mesh, J Oral Maxillofac Surg 73(7) (2015) 1437.e1-9. |  |
| **Navigation + cutting guides** | | **2** |
|  | [1] P. Juergens, Z. Krol, H.F. Zeilhofer, J. Beinemann, K. Schicho, R. Ewers, C. Klug, Computer simulation and rapid prototyping for the reconstruction of the mandible, J Oral Maxillofac Surg 67(10) (2009) 2167-70. |  |
|  | [2] S.G. Brouwer de Koning, F. Geldof, R.L.P. van Veen, M.J.A. van Alphen, L.H.E. Karssemakers, J. Nijkamp, W.H. Schreuder, T.J.M. Ruers, M.B. Karakullukcu, Electromagnetic surgical navigation in patients undergoing mandibular surgery, Sci Rep 11(1) (2021) 4657. |  |
| **Navigation + cutting guides + prebent surgical plates** | | **7** |
|  | [1] R.B. Bell, Computer planning and intraoperative navigation in cranio-maxillofacial surgery, Oral Maxillofac Surg Clin North Am 22(1) (2010) 135-56. |  |
|  | [2] R.B. Bell, K.A. Weimer, E.J. Dierks, M. Buehler, J.E. Lubek, Computer planning and intraoperative navigation for palatomaxillary and mandibular reconstruction with fibular free flaps, J Oral Maxillofac Surg 69(3) (2011) 724-32. |  |
|  | [3] Y. Shen, J. Sun, J. Li, T. Ji, M.M. Li, A revised approach for mandibular reconstruction with the vascularized iliac crest flap by virtual surgical planning, Plast Reconstr Surg 129(3) (2012) 565e-566e. |  |
|  | [4] P. Li, M. Xuan, C. Liao, W. Tang, X.Y. Wang, W. Tian, J. Long, Application of Intraoperative Navigation for the Reconstruction of Mandibular Defects With Microvascular Fibular Flaps-Preliminary Clinical Experiences, J Craniofac Surg 27(3) (2016) 751-5. |  |
|  | [5] W.B. Zhang, Y. Yu, Y. Wang, C. Mao, X.J. Liu, C.B. Guo, G.Y. Yu, X. Peng, Improving the accuracy of mandibular reconstruction with vascularized iliac crest flap: Role of computer-assisted techniques, J Craniomaxillofac Surg 44(11) (2016) 1819-1827. |  |
|  | [6] C. Yang, S. Shen, J. Wu, S. Zhang, A New Modified Method for Accurate Mandibular Reconstruction, J Oral Maxillofac Surg 76(8) (2018) 1816-1822. |  |
|  | [7] Y. Ni, X. Zhang, Z. Meng, Z. Li, S. Li, Z.F. Xu, C. Sun, F. Liu, W. Duan, Digital navigation and 3D model technology in mandibular reconstruction with fibular free flap: A comparative study, J Stomatol Oral Maxillofac Surg 122(4) (2021) e59-e64. |  |
| **Navigation + cutting guides + positioning guides + prebent surgical plates** | | **2** |
|  | [1] T. Bao, J. He, C. Yu, W. Zhao, Y. Lin, H. Wang, J. Liu, H. Zhu, Utilization of a pre-bent plate-positioning surgical guide system in precise mandibular reconstruction with a free fibula flap, Oral Oncol 75 (2017) 133-139. |  |
|  | [2] Y. Yu, W.B. Zhang, X.J. Liu, C.B. Guo, G.Y. Yu, X. Peng, Selection of Guiding Plate Combined With Surgical Navigation for Microsurgical Mandibular Reconstruction, J Craniofac Surg 31(4) (2020) 960-965. |  |
| **Navigation + positioning guides** | | **1** |
|  | [1] J.W. Huang, X.F. Shan, X.G. Lu, Z.G. Cai, Preliminary clinic study on computer assisted mandibular reconstruction: the positive role of surgical navigation technique, Maxillofac Plast Reconstr Surg 37(1) (2015) 20. |  |
| **Navigation + prebent surgical plates** | | **3** |
|  | [1] Y. Yu, W.B. Zhang, Y. Wang, X.J. Liu, C.B. Guo, X. Peng, A Revised Approach for Mandibular Reconstruction With the Vascularized Iliac Crest Flap Using Virtual Surgical Planning and Surgical Navigation, J Oral Maxillofac Surg 74(6) (2016) 1285.e1-1285.e11. |  |
|  | [2] L. Zheng, X. Lv, J. Zhang, S. Liu, J. Zhang, Y. Zhang, Translating Computer-Aided Design and Surgical Planning Into Successful Mandibular Reconstruction Using a Vascularized Iliac-Crest Flap, J Oral Maxillofac Surg 76(4) (2018) 886-893. |  |
|  | [3] D. Sozzi, A. Filippi, G. Canzi, E. De Ponti, A. Bozzetti, G. Novelli, Surgical Navigation in Mandibular Reconstruction: Accuracy Evaluation of an Innovative Protocol, J Clin Med 11(7) (2022). |  |
| **Positioning guides (only)** | | **2** |
|  | [1] X.J. Liu, L. Gui, C. Mao, X. Peng, G.Y. Yu, Applying computer techniques in maxillofacial reconstruction using a fibula flap: a messenger and an evaluation method, J Craniofac Surg 20(2) (2009) 372-7. |  |
|  | [2] V. Reiser, M. Alterman, A. Shuster, S. Kleinman, B. Shlomi, R. Yanko-Arzi, A. Zaretski, A. Amir, D.M. Fliss, V-stand--a versatile surgical platform for oromandibular reconstruction using a 3-dimensional virtual modeling system, J Oral Maxillofac Surg 73(6) (2015) 1211-26. |  |
| **Prebent surgical plates (only)** | | **13** |
|  | [1] J.W. Lee, J.J. Fang, L.R. Chang, C.K. Yu, Mandibular defect reconstruction with the help of mirror imaging coupled with laser stereolithographic modeling technique, J Formos Med Assoc 106(3) (2007) 244-50. |  |
|  | [2] C. Toro, M. Robiony, F. Costa, N. Zerman, M. Politi, Feasibility of preoperative planning using anatomical facsimile models for mandibular reconstruction, Head Face Med 3 (2007) 5. |  |
|  | [3] H.T. Cheng, C.I. Wu, C.S. Tseng, H.C. Chen, W.S. Lee, P.K. Chen, S.C. Chang, The occlusion-adjusted prefabricated 3D mirror image templates by computer simulation: the image-guided navigation system application in difficult cases of head and neck reconstruction, Ann Plast Surg 63(5) (2009) 517-21. |  |
|  | [4] A. Cohen, A. Laviv, P. Berman, R. Nashef, J. Abu-Tair, Mandibular reconstruction using stereolithographic 3-dimensional printing modeling technology, Oral Surg Oral Med Oral Pathol Oral Radiol Endod 108(5) (2009) 661-6. |  |
|  | [5] H. Essig, M. Rana, H. Kokemueller, C. von See, M. Ruecker, F. Tavassol, N.C. Gellrich, Pre-operative planning for mandibular reconstruction - a full digital planning workflow resulting in a patient specific reconstruction, Head Neck Oncol 3 (2011) 45. |  |
|  | [6] C. Rahimov, I. Farzaliyev, Virtual bending of titanium reconstructive plates for mandibular defect bridging: review of three clinical cases, Craniomaxillofac Trauma Reconstr 4(4) (2011) 223-34. |  |
|  | [7] W.H. Wang, J.Y. Deng, M. Li, J. Zhu, B. Xu, Preoperative three-dimensional reconstruction in vascularized fibular flap transfer, J Craniomaxillofac Surg 40(7) (2012) 599-603. |  |
|  | [8] L. Morris, M. Sokoya, L. Cunningham, T.J. Gal, Utility of stereolithographic models in osteocutaneous free flap reconstruction of the head and neck, Craniomaxillofac Trauma Reconstr 6(2) (2013) 87-92. |  |
|  | [9] W.H. Wang, J. Zhu, J.Y. Deng, B. Xia, B. Xu, Three-dimensional virtual technology in reconstruction of mandibular defect including condyle using double-barrel vascularized fibula flap, J Craniomaxillofac Surg 41(5) (2013) 417-22. |  |
|  | [10] N. Gao, K. Fu, J. Cai, H. Chen, W. He, The role of folded fibular flap in patients' reconstruction of mandibular defects: a retrospective clinical study, Sci Rep 11(1) (2021) 23853. |  |
|  | [11] S. Gupta, P. Goil, Formulating an Easy, Affordable, and Reproducible Method for Virtual Planning and 3D Reconstruction: A State Institution's Approach for Mandibular Reconstruction, Ann Plast Surg 87(1) (2021) 65-72. |  |
|  | [12] W.A. Odhiambo, J.M. Gatune, S.W. Guthua, C. Muraguri, Use of 3D printed model as a reconstruction aid in the management of an extensive ameloblastoma of the mandible, Clin Case Rep 10(7) (2022) e6047. |  |
|  | [13] S. Vimawala, T. Gao, J. Goldfarb, D. Gadaleta, B. Ku, P. Jean-Gilles, A. Luginbuhl, R. Pugliese, D. Weed, J.M. Curry, Initial Experience Using 3-Dimensional Printed Models for Head and Neck Reconstruction in Haiti, Ear Nose Throat J 101(3) (2022) Np89-np91. |  |
| **Prebent surgical plates (registered positions) (only)** | | **2** |
|  | [1] W. Hallermann, S. Olsen, T. Bardyn, F. Taghizadeh, A. Banic, T. Iizuka, A new method for computer-aided operation planning for extensive mandibular reconstruction, Plast Reconstr Surg 117(7) (2006) 2431-7. |  |
|  | [2] F. Wilde, M. Plail, C. Riese, A. Schramm, K. Winter, Mandible reconstruction with patient-specific pre-bent reconstruction plates: comparison of a transfer key method to the standard method--results of an in vitro study, Int J Comput Assist Radiol Surg 7(1) (2012) 57-63. |  |
| **PSSPs (only)** | | **2** |
|  | [1] Y.Y. Jo, S.G. Kim, M.K. Kim, S.H. Shin, J. Ahn, H. Seok, Mandibular Reconstruction Using a Customized Three-Dimensional Titanium Implant Applied on the Lingual Surface of the Mandible, J Craniofac Surg 29(2) (2018) 415-419. |  |
|  | [2] B.M. Barton, J.M. Blumberg, S.N. Patel, Near-total mandibular reconstruction following osteoradionecrosis with double scapula tip free flap: A case report, Microsurgery (2021). |  |
| **Virtual surgical planning (only)** | | **7** |
|  | [1] A. Eckardt, G.R. Swennen, Virtual planning of composite mandibular reconstruction with free fibula bone graft, J Craniofac Surg 16(6) (2005) 1137-40. |  |
|  | [2] V. Valentini, A. Agrillo, A. Battisti, P. Gennaro, L. Calabrese, G. Iannetti, Surgical planning in reconstruction of mandibular defect with fibula free flap: 15 patients, J Craniofac Surg 16(4) (2005) 601-7. |  |
|  | [3] K. Thankappan, N.P. Trivedi, P. Subash, S.K. Pullara, S. Peter, M.A. Kuriakose, S. Iyer, Three-dimensional computed tomography-based contouring of a free fibula bone graft for mandibular reconstruction, J Oral Maxillofac Surg 66(10) (2008) 2185-92. |  |
|  | [4] X. Yang, J. Hu, S. Zhu, X. Liang, J. Li, E. Luo, Computer-assisted surgical planning and simulation for condylar reconstruction in patients with osteochondroma, Br J Oral Maxillofac Surg 49(3) (2011) 203-8. |  |
|  | [5] C.R. Rahimov, I.M. Farzaliyev, H.R. Fathi, M.M. Davudov, A. Aliyev, E. Hasanov, The Application of Virtual Planning and Navigation Devices for Mandible Reconstruction and Immediate Dental Implantation, Craniomaxillofac Trauma Reconstr 9(2) (2016) 125-33. |  |
|  | [6] E. Marchiano, J.R. Stevens, E. Liao, A.J. Rosko, A.R. Powell, S.B. Chinn, C.L. Stucken, M.E. Spector, Three-dimensional modeling of the scapular tip for anterolateral and lateral mandibular defects, Oral Oncol 107 (2020) 104718. |  |
|  | [7] N. Callahan, M. Patel, D. Dyalram, J.E. Lubek, Is the prevention of condylar sag with maxillomandibular fixation the key to functional reconstruction of a mandibular disarticulation resection?, Oral Surg Oral Med Oral Pathol Oral Radiol 134(3) (2022) 317-322. |  |

**Supplementary Table 3.** The spatial deviation (mm) of condylar head in each case.

| Case No. | Parameter | Δx | Δy | Δz | ΔT |
| --- | --- | --- | --- | --- | --- |
| 1 | Study group | 0.32 | 0.80 | 0.42 | 0.96 |
|  | Control group | 4.31 | -2.56 | -0.17 | 5.02 |
| 2 | Study group | -1.82 | 3.24 | -0.94 | 3.83 |
|  | Control group | -2.33 | 1.05 | -0.95 | 2.73 |
| 3 | Study group | -0.54 | 0.54 | 1.43 | 1.62 |
|  | Control group | -5.18 | -1.78 | 052 | 5.50 |
| 4 | Study group | -1.78 | -0.26 | 0.08 | 1.80 |
|  | Control group | 7.04 | 2.34 | 0.86 | 7.47 |
| 5 | Study group | 3.7 | -2.59 | 2.93 | 5.38 |
|  | Control group | 4.71 | 0.05 | 0.3 | 4.72 |
| 6 | Study group | -1.77 | 1.31 | -0.53 | 2.26 |
|  | Control group | 5.02 | 0.97 | 1.47 | 5.32 |

Δx, Δy and Δz represents the deviation in X, Y, and Z directions respectively, and ΔT represents the total spatial error.

**Supplementary Table 4.** The spatial deviation (mm) of mandibular angle point in each case.

| Case No. | Parameter | Δx | Δy | Δz | ΔT |
| --- | --- | --- | --- | --- | --- |
| 1 | Study group | 1.10 | -0.02 | 1.01 | 1.49 |
|  | Control group | 6.41 | 0.45 | -2.55 | 6.91 |
| 2 | Study group | -0.82 | 2.32 | 0.65 | 2.55 |
|  | Control group | -3.47 | 2.54 | 0.69 | 4.35 |
| 3 | Study group | -1.41 | 0.45 | 1.49 | 2.10 |
|  | Control group | -2.19 | 3.55 | -2.36 | 4.79 |
| 4 | Study group | -0.35 | 0.62 | -2.12 | 2.24 |
|  | Control group | 8.84 | -9.15 | 4.29 | 13.43 |
| 5 | Study group | 1.87 | -5.23 | -1.42 | 5.73 |
|  | Control group | 4.65 | -6.11 | -0.69 | 7.71 |
| 6 | Study group | -3.44 | -3.52 | -1.81 | 5.24 |
|  | Control group | 5.64 | -3.72 | -1.33 | 6.89 |

Δx, Δy and Δz represents the deviation in X, Y, and Z directions respectively, and ΔT represents the total spatial error.

**Supplementary Table 5.** Spatial deviations for Case 1 (defect Type I).

| Parameters | Study group | Control group |
| --- | --- | --- |
| Absolute distance deviation (mm) | 0.94 | 1.69 |
| Intercondylar length (mm) | 0.21 | 4.27 |
| Intergonial length (mm) | 1.00 | 6.38 |
| Coronal mandibular angle (°) (right) | 1.96 | 3.40 |
| Coronal mandibular angle (°) (left) | 0.59 | 1.09 |
| Sagittal mandibular angle (°) (right) | 2.45 | 6.23 |
| Sagittal mandibular angle (°) (left) | 0.73 | 0.96 |
| Axial mandibular angle (°) (right) | 0.83 | 3.98 |
| Axial mandibular angle (°) (left) | 1.70 | 3.76 |

**Supplementary Table 6.** Spatial deviations for Case 2 (defect Type I).

| Parameters | Study group | Control group |
| --- | --- | --- |
| Absolute distance deviation (mm) | 0.54 | 0.96 |
| Intercondylar length (mm) | 2.06 | 2.51 |
| Intergonial length (mm) | 0.95 | 3.61 |
| Coronal mandibular angle (°) (right) | 3.36 | 5.37 |
| Coronal mandibular angle (°) (left) | 2.33 | 0.57 |
| Sagittal mandibular angle (°) (right) | 4.26 | 2.01 |
| Sagittal mandibular angle (°) (left) | 1.27 | 1.32 |
| Axial mandibular angle (°) (right) | 3.92 | 6.46 |
| Axial mandibular angle (°) (left) | 2.27 | 1.76 |

**Supplementary Table 7.** Spatial deviations for Case 3 (defect Type II).

| Parameters | Study group | Control group |
| --- | --- | --- |
| Absolute distance deviation (mm) | 0.74 | 1.99 |
| Intercondylar length (mm) | 0.56 | 5.14 |
| Intergonial length (mm) | 1.55 | 2.14 |
| Coronal mandibular angle (°) (right) | 1.20 | 6.91 |
| Coronal mandibular angle (°) (left) | 2.20 | 2.60 |
| Sagittal mandibular angle (°) (right) | 0.75 | 1.71 |
| Sagittal mandibular angle (°) (left) | 1.25 | 5.78 |
| Axial mandibular angle (°) (right) | 2.01 | 7.56 |
| Axial mandibular angle (°) (left) | 1.43 | 0.48 |

**Supplementary Table 8.** Spatial deviations for Case 4 (defect Type III).

| Parameters | Study group | Control group |
| --- | --- | --- |
| Absolute distance deviation (mm) | 1.79 | 3.10 |
| Intercondylar length (mm) | 1.77 | 7.13 |
| Intergonial length (mm) | 2.14 | 8.07 |
| Coronal mandibular angle (°) (right) | 1.31 | 3.36 |
| Coronal mandibular angle (°) (left) | 1.85 | 4.57 |
| Sagittal mandibular angle (°) (right) | 3.17 | 5.08 |
| Sagittal mandibular angle (°) (left) | 1.00 | 1.23 |
| Axial mandibular angle (°) (right) | 2.18 | 9.24 |
| Axial mandibular angle (°) (left) | 2.52 | 7.29 |

**Supplementary Table 9.** Spatial deviations for Case 5 (defect Type I).

| Parameters | Study group | Control group |
| --- | --- | --- |
| Absolute distance deviation (mm) | 0.95 | 1.91 |
| Intercondylar length (mm) | 3.77 | 4.72 |
| Intergonial length (mm) | 2.14 | 4.98 |
| Coronal mandibular angle (°) (right) | 2.51 | 1.67 |
| Coronal mandibular angle (°) (left) | 2.00 | 2.85 |
| Sagittal mandibular angle (°) (right) | 0.38 | 6.05 |
| Sagittal mandibular angle (°) (left) | 2.13 | 2.78 |
| Axial mandibular angle (°) (right) | 4.01 | 8.07 |
| Axial mandibular angle (°) (left) | 1.60 | 1.63 |

**Supplementary Table 9.** Spatial deviations for Case 6 (defect Type I).

| Parameters | Study group | Control group |
| --- | --- | --- |
| Absolute distance deviation (mm) | 1.19 | 3.19 |
| Intercondylar length (mm) | 1.82 | 5.07 |
| Intergonial length (mm) | 3.39 | 5.69 |
| Coronal mandibular angle (°) (right) | 0.60 | 4.50 |
| Coronal mandibular angle (°) (left) | 0.53 | 4.97 |
| Sagittal mandibular angle (°) (right) | 2.42 | 10.65 |
| Sagittal mandibular angle (°) (left) | 2.11 | 6.48 |
| Axial mandibular angle (°) (right) | 3.56 | 3.82 |
| Axial mandibular angle (°) (left) | 1.54 | 6.19 |
